# On the qualitative study of a discrete-time phytoplankton-zooplankton model under the effects of external toxicity in phytoplankton population

**DOI:** 10.1016/j.heliyon.2022.e12415

**Published:** 2022-12-16

**Authors:** Muhammad Salman Khan, Maria Samreen, J.F. Gómez-Aguilar, Eduardo Pérez-Careta

**Affiliations:** a Department of Mathematics, Quaid-I-Azam University, Islamabad, 44230, Pakistan; b CONACyT-Tecnológico Nacional de México/CENIDET, Interior Internado Palmira S/N, Col. Palmira, C.P. 62490, Cuernavaca, Morelos, Mexico; c Universidad de Guanajuato, Dpto. Electrónica, Carretera Salamanca-Valle de Santiago, Km 3+1.8, Salamanca Gto, Mexico

**Keywords:** Phytoplankton-zooplankton model, Holling type-II response, Euler's forward method, Nonstandard finite difference scheme, Boundedness, Stability analysis, Neimark-Sacker bifurcation, Period-doubling bifurcation, Generalized hybrid control methodology

## Abstract

The current manuscript studies a discrete-time phytoplankton-zooplankton model with Holling type-II response. The original model is modified by considering the condition that the phytoplankton population is getting infected with an external toxic substance. To obtain the discrete counterpart from a continuous-time system, Euler's forward method is applied. Moreover, a consistent discrete-time phytoplankton-zooplankton model is obtained by using a nonstandard difference scheme. The boundedness character for every positive solution is discussed, and the local stability of obtained system about each of its fixed points is discussed. The existence of period-doubling bifurcation at a positive equilibrium point is discussed for the discrete system obtained by Euler's forward method. In addition, the comparison of the consistent discrete-time version with its inconsistent counterpart is provided. It is proved that the discrete-time system obtained by using a nonstandard scheme is dynamically consistent as there is no chance for the existence of period-doubling bifurcation in that system. In order to control the period-doubling bifurcation and Neimark-Sacker bifurcation, an improved hybrid control strategy is applied. Finally, we have provided some interesting numerical examples to explain our theoretical results.

## 1 Introduction

Many scientific investigations have focused on mathematical modelling and studying population dynamics over the last two decades. Moreover, in that period, some well-known mathematical models in abstract ecology have been presented, and the most famous one is the Lotka-Volterra model [1]. The study of creature movement and scattering has turned out to be a vital element for understanding a sequence of environmental interrogations connected with the spatiotemporal analysis of population dynamics [2]. Plankton is enormously flexible in abundance, both temporally and spatially. Plankton discrepancy depends on the regular and the spatial structure's physical procedure. Biological processes include, for instance, growth, eating and behaviour. Moreover, physical processes are involved as adjacent stirring. The nonlinearity of bio networks contributes entirely to the spatial union in plankton distributions [3]. In marine ecology, the word plankton refers to a class of freely moving and hazily swimming organisms. Generally, plankton is of two kinds: phytoplankton and zooplankton. Phytoplankton classes have a petite size with a single-celled structure [4]. By primary formation, sinking and decease, they put forth a worldwide effect on the environment by efficiently carrying $CO_{2}$ from the ocean's exterior layer to the deep aquatic sediments. The algal species grow in large quantities in marine and limnic atmospheres. The stages of enhanced development slowed stagnation and rapid degeneration of cell counts collectively creating an algal bloom. This type of rapid variation in the phytoplankton population density is a uniqueness in the plankton ecosystem [4]. Even though the unexpected appearing and fading of blooms are not well understood, the opposing properties of damaging algal blooms on the health of humanity, marine population, fisheries industry, and tourism are proven (see [4]). The analysis of the interaction of phytoplankton and zooplankton on the happening of bloom is of interest to several scientific studies [5]. Phytoplankton creates venoms to evade the predation by zooplankton. Decrease of eating force of zooplankton in arrears to the ejection of venomous materials by phytoplankton is one of the motivating topics of investigation in the past years. The venom making phytoplankton decreases the grazing force on them. Thoughtful of the dynamics of plankton inhabitants and their relations are of primary significance. Numerous phytoplankton species are poisonous to zooplankton. This poisonousness affects the spreading of phytoplankton and zooplankton populations. Scientific modelling of plankton population is an adequate substitute procedure in enlightening our information about the biological and physical processes connecting to the plankton ecosystem [5]. In [6], the authors have discussed a plankton-nutrient system related to marine ecology by examining planktonic blooms. The authors in [7] have discussed the effects of seasonality on the plankton population and explored the impact of periodicity on their dynamical behaviour.

By taking the intense depiction of viral diseased phytoplankton and infections, the author, in [8] has developed and studied two different mathematical models for the plankton population. The co-occurrence of competitive predators and possible extinction of prey due to the effects of predation are discussed in [9]. Above and beyond, they have studied a two-zooplankton one-phytoplankton model together through the observation of harvesting.

A nutrient-phytoplankton model is investigated in [10] to scrutinize the dynamics of phytoplankton blooms. Yunfei et al. [11] have presented a biological model for zooplankton-phytoplankton dynamics by including an extra condition of harvesting of populations. In addition, they have explained that over-manipulation may act as a source of the destruction of the population; however, proper harvesting must confirm the defensibility of the population. Similarly, several researchers have studied the dynamics of phytoplankton-zooplankton interacting systems by using the co-occurrence of plankton, a nutrient cause, the influence of harvesting, or the vicious influence of the plankton system [9], [10], [11], [12], [13], [14], [15]. Chattopadhyay and Sarkar [16] have presented and studied a mathematical model by considering the time delay in toxin escape by phytoplankton. In addition, it is suitable to lead with the poison-producing delay throughout the learning of the dynamical behaviour of phytoplankton-zooplankton population models. The efforts made in [17], [18], [19], [20], [21], [22], [23] inspired us to study and discuss the dynamical behaviour of a phytoplankton-zooplankton inhabitant's model using poisonousness. Additionally, this poisonous material is released by phytoplankton and sometimes by an external toxic source. For the study of some interesting models in mathematical ecology and mathematical chemistry, we refer a reader to [24], [25], [26], [27]. Here, we have considered the basic idea of modelling phytoplankton-zooplankton interactions, which is taken from Zhang and Rehim [28]. Moreover, while studying our mathematical model, we focus on the following situations.

• We consider that p(t) and z(t) are densities of zooplankton and phytoplankton populations at any time *t*.
• The zooplankton population is continuously consuming the phytoplankton population and recycles them into their community.
• We have considered that the zooplankton population becomes diseased by ingesting the diseased phytoplankton population. Furthermore, the contagion in phytoplankton may produce due to some external toxic substances (see [28]).
• The phytoplankton has exponential growth in the nonappearance of the zooplankton population. Where *r* is their logistic rate of change, and *k* is the maximum carrying capacity of the surroundings [29].
• We have taken that the time lag for phytoplankton's creation and mediation of toxic stuff is zero.

Under these conditions, as mentioned earlier, we have the following phytoplankton-zooplankton model from [28]:

(1) $$\frac{dp}{dt}=rp(t)-\frac{rp^{2}(t)}{k}-\alpha z(t)f(p(t)),\frac{dz}{dt}=\beta z(t)f(p(t))-\rho z(t)g(p(t))-\delta z(t).$$

Kaung [30] examined the behaviour of a prey-predator model by using the Holling type-II response function. Moreover, he explained that studying the dynamical assets of prey-predator models using Holling type response is preferable to studying dynamics of predator-prey models without using Holling response [31]. Generally, we define the Holling type-II response function by the basic definition of rectangular hyperbola, and its mathematical form is:

$$\varphi (y)=\frac{y}{\alpha +y}.$$

Where *α* is some constant, by using Holling type-II response [31], we have the next mathematical shape of the system (1):

(2) $$\frac{dp}{dt}=rp(t)-\frac{rp^{2}(t)}{k}-\alpha z(t)\frac{p(t)}{a+p(t)}-mp^{2}(t),\frac{dz}{dt}=\beta z(t)\frac{p(t)}{a+p(t)}-\rho z(t)\frac{p(t)}{a+p(t)}-\delta z(t).$$

• The functional response $\frac{\alpha p(t)z(t)}{a+p(t)}$ characterizes the rate at which phytoplankton population is eaten by zooplankton. In addition, it causes a rise in the development rate of zooplankton and this development rate is represented by $\frac{\beta p(t)z(t)}{a+p(t)}$.
• The term $mp^{2}(t)$ in the system (2) shows that the infection developed in the phytoplankton population is due to external toxic substances.

Additionally, the term $\frac{d^{2}}{dp^{2}}(mp^{2})=2>0$ illustrates the fast-tracking growth of poisonous substance corresponding to phytoplankton population, as approximately every type of phytoplankton population is increasingly consuming toxic substances. Where the parametric values in the system (2) are non-negative and defined along these lines:

• *α*: the highest seizure rate of zooplankton on phytoplankton.
• *a*: the constant of fractional catching saturation.
• *β*: the transformation rate of phytoplankton-zooplankton (β<α).
• *ρ*: the poisonousness rate of phytoplankton for every unit biomass.
• *δ*: the decease rate of zooplankton inhabitants.

According to Strogatz [32], chaos occurs in a continuous system when it is at least 3-dimensional. Therefore, it is clear that chaos ceased to exist in the system (2). However, in the case of a discrete-time map, chaos can be observed in 1-dimension. Hence, there is a chance for the existence of chaos in the 2-dimensional discrete-time phytoplankton-zooplankton model. In addition, if chaos does not exist after the discretization then the discretized system is said to be dynamically consistent. Motivated by the aforementioned prosperous characteristics of discrete-time dynamical systems, it is necessary and exciting to study the qualitative behaviour of the discrete-time version of the system (2). Moreover, our main aim is to study a consistent counterpart of the system (2) such that there is a minimal change in the dynamical behaviour of the discretized system compared to the original continuous system. Therefore, by using Euler's forward method with step size *η*, we have the following discrete-time version of (2):

(3) $$p_{n+1}=p_{n}+\eta \left(rp_{n}(1-\frac{p_{n}}{k})-\frac{\alpha p_{n}z_{n}}{a+p_{n}}-mp_{n}^{2}\right),z_{n+1}=z_{n}+\eta \left(\frac{\beta p_{n}z_{n}}{a+p_{n}}-\delta z_{n}-\frac{\rho p_{n}z_{n}}{a+p_{n}}\right).$$

Moreover, to obtain a consistent counterpart of system (3) and by applying the Micken's type nonstandard scheme on the model (3), we get the following discrete-time mathematical model (see [33]):

(4) $$p_{n+1}=\frac{(1+\eta r)p_{n}}{1+\eta \left(\frac{r}{k}p_{n}+\frac{\alpha z_{n}}{a+p_{n}}+mp_{n}\right)},z_{n+1}=\frac{\left(1+\frac{\eta \beta p_{n}}{a+p_{n}}\right)z_{n}}{1+\eta \left(\delta +\frac{\rho p_{n}}{a+p_{n}}\right)}.$$

The next part of this manuscript is structured along these lines:

• The boundedness character for every positive solution of system (4) is discussed in section 2.
• The existence of fixed points and local stability of system (3) and (4) about each of them is investigated in section 3.
• The presence of Neimark-Sacker bifurcation about the unique positive fixed point of system (4) is discussed in section 4.
• The existence of period-doubling bifurcation about one and only positive fixed point of system (3) is discussed in section 5.
• We discussed a modified hybrid control strategy for controlling the chaos, period-doubling bifurcation and Neimark-Sacker bifurcation in section 6.
• A comprehensive numerical simulation is provided in section 7 to support each theoretical investigation.

## 2 Boundedness of solutions of system (4)

We assume that $p_{0}>0$ and $z_{0}>0$, with $z_{n}\geq p_{n}$ for all n≥0. Then every solution $\left(p_{n},z_{n}\right)$ of the system (4) must fulfils $p_{n}>0$ and $z_{n}>0$ for all n≥0. Moreover, from first part of (4) we get

(5) $$p_{n+1}\leq \frac{(1+\eta r)p_{n}}{1+\eta p_{n}(\frac{r}{k}+m)}.$$

On solving (5), and by applying limit we get

(6) $$\lim_{n⟶\infty }supp_{n}\leq \frac{kr}{km+r},$$

for every n≥0. In addition, for $z_{n}\geq p_{n}$ for all n≥0, from second part of system (4), we get

$$z_{n+1}\leq \frac{\left(1+\frac{\eta \beta p_{n}}{a+p_{n}}\right)z_{n}}{1+\eta \left(\delta +\frac{\rho z_{n}}{a+p_{n}}\right)},$$

$$\leq \frac{\left(1+\frac{\eta \beta (\frac{kr}{km+r})}{a+(\frac{kr}{km+r})}\right)z_{n}}{1+\eta \left(\delta +\frac{\rho z_{n}}{a+(\frac{kr}{km+r})}\right)}.$$

Hence, one can obtain the upper bound for zooplankton population as

(7) $$\lim_{n\to \infty }supz_{n}\leq \frac{kr(\beta -\delta -\rho )-a\delta (km+r)}{\rho (km+r)},$$

for all n≥0. Finally, we have the following theorem about the boundedness of all solutions of (4).

Theorem 2.1 *Assume that*$z_{n}\geq p_{n}$*for all*n≥0*, with*$0<p_{0}\leq \frac{kr}{km+r}$*and*$0<z_{0}\leq \frac{kr(\beta -\delta -\rho )-a\delta (km+r)}{\rho (km+r)}$*, then for all*n≥0*, each positive solution*$\left(p_{n},z_{n}\right)$*of system*(4)*is bounded and contained in the set*$\left[0,\frac{kr}{km+r}\right]\times \left[0,\frac{kr(\beta -\delta -\rho )-a\delta (km+r)}{\rho (km+r)}\right]$*, whenever*β>ρ+δ*and*kr(β−δ−ρ)>aδ(km+r)*.*

## 3 Stability analysis of fixed points of system (4)

Assume that, $p_{n+1}=p_{n}=\overset{¯}{p}$ and $z_{n+1}=z_{n}=\overset{¯}{z}$. Then from system (3) and (4) we get three fixed points, $\bar{o}=(0,0)$, $\bar{b}=(\frac{kr}{km+r},0)$ and $\bar{x}=\left(\frac{a\delta }{\beta -\delta -\rho },\frac{a(\beta -\rho )((kr(\beta -\delta -\rho )-akm\delta )-ar\delta )}{k\alpha {\left(-\beta +\delta +\rho \right)}^{2}}\right)$. Moreover, $\bar{x}$ represents the positive fixed point of system (4) if we have

(8) β>δ+ρandkr(β−δ−ρ)>aδ(km+r).

In order to discuss the stability of fixed points, we have the following lemma from [34].

Lemma 3.1 *[34]**Let*

(9) $$F(\omega )=\omega^{2}-T\omega +D$$

*be the characteristic equation obtained from a*2×2*jacobian matrix J. Moreover, J be the jacobian matrix of system*(3)*or*(4)*about each of its equilibrium point. Additionally, assume that T and D are respectively trace and determinant of J and*F(1)>0*. Then:*

1. $|\omega_{1}|<1$*and*$|\omega_{2}|<1$*if and only if*F(−1)>0*and*D<1*.*
2. $|\omega_{1}|>1$*and*$|\omega_{2}|>1$*if and only if*F(−1)>0*and*D>1*.*
3. $|\omega_{1}|<1$*and*$|\omega_{2}|>1$*or*$(|\omega_{1}|>1$*and*$\left|\omega_{2}<|1\right)$*if and only if*F(−1)<0*.*
4. $\omega_{1}=-1$*and*$|\omega_{2}|\neq 1$*if and only if*F(−1)=0*and*D≠1,−1*.*
5. $\omega_{1}$*and*$\omega_{2}$*represent complex conjugates with*$\left|\omega_{1}|=1=|\omega_{2}\right|$*if and only if*$T^{2}-4D<0$*and*D=1*.*

*As*$\omega_{1}$*and*$\omega_{2}$*are eigenvalues of*(9)*then we have the subsequent topological type outcomes related to the stability of*$\underline{p}$*. Where p be any arbitrary fixed point of*(9)*. The point*$\underline{p}$*is identified as sink if*$|\omega_{1}|<1$*and*$|\omega_{2}|<1$*, it is locally asymptotically stable. The point*$\underline{p}$*is known as source if*$|\omega_{1}|>1$*and*$|\omega_{2}|>1$*, as source is repeller hence it remains unstable. The point*$\underline{p}$*is known as saddle if*$|\omega_{1}|<1$*and*$|\omega_{2}|>1$*or*$\left(|\omega_{1}|>1\;and\;|\omega_{2}|<1\right)$*. The point*$\underline{p}$*is acknowledged as non-hyperbolic if either 4 or 5 is satisfied.*

Firstly, we consider the fixed point $\bar{o}=(0,0)$ of system (4), then we have the following result about its stability

Proposition 3.1 *Let*

(10) $$J[0,0]=\left[\begin{array}{cc} 1+r\eta \; & 0\\ 0\; & \frac{1}{1+\delta \eta } \end{array}\right]$$

*be the jacobian matrix of system*(4)*about*(0,0)*. Then, the trivial fixed point*$\bar{o}$*of system*(4)*remains a saddle point as*|1+rη|>1*and*$\frac{1}{\left|1+r\delta \right|}<1$*for every*r,η,δ>0*.*

Next, by considering the fixed point $\left(\frac{kr}{km+r},0\right)$ of the system (4) we get the following result:

Proposition 3.2 *Let*

(11) $$J[\frac{kr}{km+r},0]=\left[\begin{array}{cc} \frac{1}{1+r\eta }\; & -\frac{kr\alpha \eta }{\left(akm+(a+k)r)(1+r\eta \right)}\\ 0\; & \frac{a(km+r)+kr(1+\beta \eta )}{(akm+(a+k)r)(1+\delta \eta )+kr\eta \rho } \end{array}\right]$$

*be the jacobian matrix of system*(4)*about*$\left(\frac{kr}{km+r},0\right)$*. Then, the fixed point*$\left(\frac{kr}{km+r},0\right)$*of system*(4)*will be classified as*

• *Sink: if and only if we have*krβ<aδ(km+r)+kr(δ+ρ)*.*
• *Saddle: if and only if we have*krβ>aδ(km+r)+kr(δ+ρ)*.*

**Figure 1:**
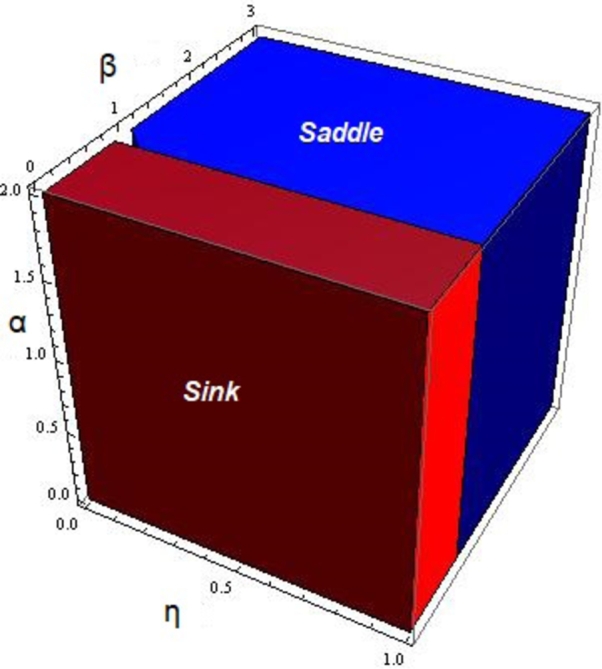
Topological classification of fixed point $\left(\frac{kr}{km+r},0\right)$ for 0 < *η* < 1, 0 < *α* < 2, 0 < *β* < 3, *r* = 2.4917,*m* = 0.217,*δ* = 0.32,*a* = 0.217,*k* = 0.217 and *ρ* = 0.217.

In Fig. 1 the topological classification of fixed point $\left(\frac{kr}{km+r},0\right)$ for some values of parameters is given.

Now, we assume that (8) remains true, then by considering the fixed point $\bar{x}$ of system (4) we have

$$J[\bar{x}]=\left[\begin{array}{cc} 1-\frac{\delta \eta (a(km+r)(\beta +\delta -\rho )+kr(-\beta +\delta +\rho ))}{k(1+r\eta )(\beta -\rho )(\beta -\delta -\rho )}\; & \frac{\alpha \delta \eta }{\left(1+r\eta )(-\beta +\rho \right)}\\ \frac{\eta (\beta -\rho )(-ar\delta -k(am\delta +r(-\beta +\delta +\rho )))}{k\alpha (\beta +\beta \delta \eta -\rho )}\; & 1 \end{array}\right].$$

Moreover, the characteristic polynomial obtained from matrix $J[\bar{x}]$ is given as follows:

(12) $$U(\xi )=\xi^{2}-\xi T+D,$$

where,

$$T=2-\frac{\delta \eta (a(km+r)(\beta +\delta -\rho )+kr(-\beta +\delta +\rho ))}{k(1+r\eta )(\beta -\rho )(\beta -\delta -\rho )},$$

and

$$D=\frac{1}{1+r\eta }\left(1+r\eta +r\delta \eta^{2}+\frac{(akm+(a+k)r)\delta \eta }{k(\beta -\rho )}-\frac{\delta^{2}\eta^{2}(a(km+r)+kr(1+\beta \eta ))}{k(\beta +\beta \delta \eta -\rho )}-\frac{2a(km+r)\delta \eta }{k(\beta -\delta -\rho )}\right).$$

By assuming that (8) remains true and by performing some mathematical operations, it follows that:

(13) $$U(1)=\frac{\delta \eta^{2}(rk(\beta -\delta -\rho )-a\delta (r+km))}{k(1+r\eta )(\beta +\beta \delta \eta -\rho )}>0,$$

and

(14) $$U(-1)=\frac{4+\eta \left(r(4+\delta \eta )+\frac{2(akm+(a+k)r)\delta }{k(\beta -\rho )}-\frac{\delta^{2}\eta (a(km+r)+kr(1+\beta \eta ))}{k(\beta +\beta \delta \eta -\rho )}-\frac{4a(km+r)\delta }{k(\beta -\delta -\rho )}\right)}{1+r\eta }.$$

Remark 3.1 Assume that (8) remains true, then there is no chance of period-doubling bifurcation in system (4) as U(−1)>0 for every a,m,ρ,η,δ,α,β>0.

We have the following theorem for the possible validation of Remark 3.1.

Theorem 3.1 *Let*(8)*remains true and*$\bar{x}=\left(\frac{a\delta }{\beta -\delta -\rho },\frac{a(\beta -\rho )((kr(\beta -\delta -\rho )-akm\delta )-ar\delta )}{k\alpha {\left(-\beta +\delta +\rho \right)}^{2}}\right)$*be the positive fixed point of system*(4)*and*

$$U(-1)=\frac{4+\eta \left(r(4+\delta \eta )+\frac{2(akm+(a+k)r)\delta }{k(\beta -\rho )}-\frac{\delta^{2}\eta (a(km+r)+kr(1+\beta \eta ))}{k(\beta +\beta \delta \eta -\rho )}-\frac{4a(km+r)\delta }{k(\beta -\delta -\rho )}\right)}{1+r\eta }.$$

*Then*U(−1)>0*for every*a,m,ρ,η,δ,α,β>0*.*

Proof Assume (8) and let $\left(\frac{a\delta }{\beta -\delta -\rho },\frac{a(\beta -\rho )((kr(\beta -\delta -\rho )-akm\delta )-ar\delta )}{k\alpha {\left(-\beta +\delta +\rho \right)}^{2}}\right)$ be the positive fixed point of system (4) and

$$U(-1)=\frac{4+\eta \left(r(4+\delta \eta )+\frac{2(akm+(a+k)r)\delta }{k(\beta -\rho )}-\frac{\delta^{2}\eta (a(km+r)+kr(1+\beta \eta ))}{k(\beta +\beta \delta \eta -\rho )}-\frac{4a(km+r)\delta }{k(\beta -\delta -\rho )}\right)}{1+r\eta }.$$

Then, U(−1)>0 if and only if we have

$$r(4+\delta \eta )+\frac{2\delta (akm+(a+k)r)}{k(\beta -\rho )}>\frac{\delta^{2}\eta (a(km+r)+kr(1+\beta \eta ))}{k(\beta +\beta \delta \eta -\rho )}+\frac{4a(km+r)\delta }{k(\beta -\delta -\rho )},$$

(15) $$⟹r(4+\delta \eta )+\frac{2\delta (akm+(a+k)r)}{k(\beta -\rho )}>\frac{4\delta a(km+r)}{k}\left(\frac{1}{\beta -\delta -\rho }\right)+\frac{\delta^{2}\eta r(1+\beta \eta )}{\left(\beta +\beta \delta \eta -\rho \right)}.$$

As

$$\frac{\delta^{2}\eta (a(km+r)+kr(1+\beta \eta ))}{k(\beta +\beta \delta \eta -\rho )}>\frac{4\delta a(km+r)}{k}\left(\frac{1}{\beta -\delta -\rho }\right),$$

with

β>δ+ρandkr(β−δ−ρ)>aδ(km+r),

then, we have

$$\frac{a\delta (km+r)}{kr(\beta -\delta -\rho )}<1.$$

Formally, from (15) we get

$$r(4+\eta \delta )+\frac{2\delta a(km+r)}{k(\beta -\rho )}+\frac{2r\delta }{\beta -rho}>4r+\frac{\delta^{2}\eta r(1+\beta \eta )}{\left(\beta +\beta \delta \eta -\rho \right)},$$

$$⟹\delta \eta r+\frac{2\delta }{\beta -\rho }+2>\frac{\delta^{2}\eta r(1+\beta \eta )}{\left(\beta +\beta \delta \eta -\rho \right)}$$

(16) $$⟹\delta \eta +4>\frac{\delta^{2}\eta r(1+\beta \eta )}{\left(\beta +\beta \delta \eta -\rho \right)}.$$

The inequality (16) is true for every choice of m,ρ,η,δ,a,α and β≥ρ. Which completes the proof. □

The following proposition around the local stability analysis of the system (4) about $\bar{x}$.

Proposition 3.3 *Assume that*(16)*holds true and*$J[\bar{x}]$*be the jacobian matrix of the system*(4)*about*$\bar{x}$*and*U(−1)>0*. Then, the following conditions are satisfied:*

1. *The point*$\bar{x}$*is sink if and only if*
  a(km+r)(1+δη)+krδη(1+βη)+krηρ>kr(1+βη).
2. *The point*$\bar{x}$*is source if and only if*
  a(km+r)(1+δη)+krδη(1+βη)+krηρ<kr(1+βη).
3. *The point*$\bar{x}$*undergoes the Neimark-Sacker bifurcation if and only if*
  (17) $$\eta =\frac{\left(\beta -\rho )(a(km+r)(\beta +\delta -\rho )-kr(\beta -\delta -\rho )\right)}{rk(\beta -\delta -\rho )\left(\beta^{2}-2\beta \rho +\rho (\delta +\rho )\right)-a\delta \left(2\beta^{2}-3\beta \rho +\rho (\delta +\rho )\right)(r+km)}$$
  (18) $$\left\{ \begin{array}{c} \frac{{\left(akm+(a+k)r\right)}^{2}\delta }{{\left(\beta -\rho \right)}^{2}}+\frac{4a(km+r)(akm+(a+k)r)}{\beta -\rho }+\frac{4k\delta (1+r\eta )(a(km+r)+kr(1+\beta \eta ))}{\beta +\beta \delta \eta -\rho }+\frac{4a^{2}{\left(km+r\right)}^{2}\delta }{{\left(\beta -\delta -\rho \right)}^{2}}<\\ 4k^{2}r(1+r\eta )+\frac{4a(km+r)(akm+(a+k)r)}{\beta -\delta -\rho }. \end{array}\right.$$

**Figure 2:**
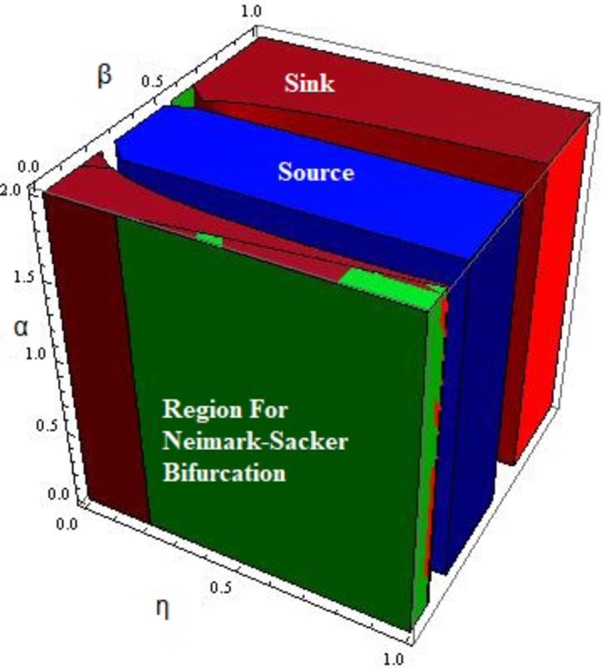
Topological classification of fixed point $\bar{x}$ for 0 < *η* < 1, 0 < *α* < 2, 0 < *β* < 1, *r* = 2.4917,*m* = 0.217,*δ* = 0.32,*a* = 0.217,*k* = 0.217 and *ρ* = 0.217.

In Fig. 2, the topological classification of fixed point $\bar{x}$ for some parametric values is given. Moreover, it can be seen that increasing the parameter *β* may cause an increase in the bifurcation region.

## 4 Neimark-Sacker bifurcation for system (4)

In this section, we study the existence of the Neimark-Sacker bifurcation about the only positive steady-state of the system (4). We have used the standard theory of bifurcation for the direction and presence of this kind of bifurcation. In recent times, Neimark-Sacker bifurcation associated with some discrete-time mathematical systems has been examined by several scientists [35], [36], [37], [38]. Additionally, when mathematical models are in differential form, we refer to [39], [40], [41] for specific considerations associated with Hopf bifurcation. First, we confirm that the positive equilibrium point $\bar{x}$ of the system (4) experiences the Neimark-Sacker bifurcation whenever *η* is chosen as a bifurcation parameter. One can see from Proposition 3.3 that roots $\xi_{1},\xi_{2}$ of (8) are complex and satisfy $|\xi_{1}|=|\xi_{2}|=1$ if and only if

$$\eta =\frac{\left(\beta -\rho )(a(km+r)(\beta +\delta -\rho )-kr(\beta -\delta -\rho )\right)}{rk(\beta -\delta -\rho )\left(\beta^{2}-2\beta \rho +\rho (\delta +\rho )\right)-a\delta \left(2\beta^{2}-3\beta \rho +\rho (\delta +\rho )\right)(r+km)},$$

and

$$\left\{ \begin{array}{c} \frac{{\left(akm+(a+k)r\right)}^{2}\delta }{{\left(\beta -\rho \right)}^{2}}+\frac{4a(km+r)(akm+(a+k)r)}{\beta -\rho }+\frac{4k\delta (1+r\eta )(a(km+r)+kr(1+\beta \eta ))}{\beta +\beta \delta \eta -\rho }+\frac{4a^{2}{\left(km+r\right)}^{2}\delta }{{\left(\beta -\delta -\rho \right)}^{2}}<\\ 4k^{2}r(1+r\eta )+\frac{4a(km+r)(akm+(a+k)r)}{\beta -\delta -\rho }. \end{array}\right.$$

Furthermore, under the supposition that (8) remains true, we have the following set:

$$\Phi_{2}=\{\alpha ,\beta ,\delta ,\rho ,r,k\in {ℜ}^{+}:\eta_{1}=\frac{\left(\beta -\rho )(a(km+r)(\beta +\delta -\rho )-kr(\beta -\delta -\rho )\right)}{rk(\beta -\delta -\rho )\left(\beta^{2}-2\beta \rho +\rho (\delta +\rho )\right)-a\delta \left(2\beta^{2}-3\beta \rho +\rho (\delta +\rho )\right)(r+km)}\}.$$

Then, the positive fixed point $\bar{x}$ of system (4) experiences the Neimark-Sacker bifurcation for parameter *η* when it varies in a slight neighbourhood of $\overset{ˆ}{\eta }$, which is given as

$$\overset{ˆ}{\eta }=\frac{\left(\beta -\rho )(a(km+r)(\beta +\delta -\rho )-kr(\beta -\delta -\rho )\right)}{rk(\beta -\delta -\rho )\left(\beta^{2}-2\beta \rho +\rho (\delta +\rho )\right)-a\delta \left(2\beta^{2}-3\beta \rho +\rho (\delta +\rho )\right)(r+km)}.$$

In addition, assume that $(\alpha ,\beta ,\delta ,\rho ,\eta ,r,k,m)\in \Phi_{2}$ then the system (4) is characterized equivalently with the following planer map:

(19) $$\left(\begin{array}{c} p\\ z \end{array}\right)\to \left(\begin{array}{c} \frac{(1+\overset{ˆ}{\eta }r)p}{1+\overset{ˆ}{\eta }\left(\frac{r}{k}p+\frac{\alpha z}{a+p}+mp\right)}\\ \frac{\left(1+\frac{\overset{ˆ}{\eta }\beta p}{a+p}\right)z}{1+\overset{ˆ}{\eta }\left(\delta +\frac{\rho pz}{a+p}\right)} \end{array}\right).$$

To discuss and analyze the normal form theory for Neimark-Sacker bifurcation for fixed point

$$\bar{x}=\left(\frac{a\delta }{\beta -\delta -\rho },\frac{a(\beta -\rho )((kr(\beta -\delta -\rho )-akm\delta )-ar\delta )}{k\alpha {\left(-\beta +\delta +\rho \right)}^{2}}\right),$$

of (28), we suppose that $\eta_{1}$ represents a small perturbation in $\overset{ˆ}{\eta }$. Then the perturbed mapping for (19) can be described by the next map:

(20) $$\left(\begin{array}{c} p\\ z \end{array}\right)\to \left(\begin{array}{c} \frac{(1+(\overset{ˆ}{\eta }+\eta_{1})r)p}{1+(\overset{ˆ}{\eta }+\eta_{1})\left(\frac{r}{k}p+\frac{\alpha z}{a+p}+mp\right)}\\ \frac{\left(1+\frac{(\overset{ˆ}{\eta }+\eta_{1})\beta p}{a+p}\right)z}{1+(\overset{ˆ}{\eta }+\eta_{1})\left(\delta +\frac{\rho pz}{a+p}\right)} \end{array}\right).$$

Taking $x=p-\frac{a\delta }{\beta -\delta -\rho }$ and $y=z-\frac{a(\beta -\rho )((kr(\beta -\delta -\rho )-akm\delta )-ar\delta )}{k\alpha {\left(-\beta +\delta +\rho \right)}^{2}}$. Then from equation (20) we get the following mapping;

(21) $$\left(\begin{array}{c} x\\ y \end{array}\right)\to \left(\begin{array}{cc} \theta_{11}\; & \theta_{12}\\ \theta_{21}\; & \theta_{22} \end{array}\right)\left(\begin{array}{c} x\\ y \end{array}\right)+\left(\begin{array}{c} h_{1}(x,y)\\ h_{2}(x,y) \end{array}\right),$$

where,

$$h_{1}(x,y)=\theta_{13}x^{2}+\theta_{14}xy+\theta_{15}y^{2}+\theta_{16}x^{3}+\theta_{17}x^{2}y+\theta_{18}xy^{2}+\theta_{19}y^{3}+O\left({\left(|x|+|y||\right)}^{4}\right),h_{2}(x,y)=\theta_{23}x^{2}+\theta_{24}xy+\theta_{25}y^{2}+\theta_{26}x^{3}+\theta_{27}x^{2}y+\theta_{28}xy^{2}+\theta_{29}y^{3}+O\left({\left(|x|+|y|\right)}^{4}\right),$$

and

$$\theta_{11}=1-\frac{\delta (\overset{ˆ}{\eta }+\eta_{1})(a(km+r)(\beta +\delta -\rho )-kr(\beta -\delta -\rho ))}{k(1+r(\overset{ˆ}{\eta }+\eta_{1}))(\beta -\rho )(\beta -\delta -\rho )},\;\theta_{12}=\frac{\alpha \delta (\overset{ˆ}{\eta }+\eta_{1})}{\left(1+r(\overset{ˆ}{\eta }+\eta_{1}))(\rho -\beta \right)},\theta_{13}=\frac{\eta \left(a^{2}{\left(km+r\right)}^{2}\delta \eta {\left(\beta +\delta -\rho \right)}^{2}+k^{2}r{\left(-\beta +\delta +\rho \right)}^{2}(\beta -\delta +r\beta \eta -(1+r\eta )\rho )\right)}{ak^{2}{\left(1+r\eta \right)}^{2}{\left(\beta -\rho \right)}^{2}(\beta -\delta -\rho )}-\frac{(km+r)\eta \left(\delta^{2}(r\eta -1)+\beta^{2}(1+r\eta )+(1+r\eta )\rho^{2}-\delta (\rho +3r\eta \rho )+\beta (\delta +3r\delta \eta -2(\rho +r\eta \rho ))\right)}{k{\left(1+r\eta \right)}^{2}{\left(\beta -\rho \right)}^{2}},\theta_{14}=-\frac{\alpha \eta \left(\beta^{2}(1+r\eta )+\delta^{2}(1-2am\eta -r\eta )+(1+r\eta )\rho^{2}+2\delta (\rho +am\eta \rho )-2\beta (\delta +am\delta \eta +\rho +r\eta \rho )\right)}{a{\left(1+r\eta \right)}^{2}{\left(\beta -\rho \right)}^{2}}+\frac{2r\alpha \delta \eta^{2}(\beta +\delta -\rho )}{k{\left(1+r\eta \right)}^{2}{\left(\beta -\rho \right)}^{2}},\;\theta_{15}=\frac{\alpha^{2}\delta \eta^{2}(\beta -\delta -\rho )}{a{\left(1+r\eta \right)}^{2}{\left(\beta -\rho \right)}^{2}},\theta_{16}=\frac{\eta^{2}{\left(a(km+r)(\beta +\delta -\rho )+kr(-\beta +\delta +\rho )\right)}^{2}}{a^{2}k^{2}{\left(1+r\eta \right)}^{2}{\left(\beta -\rho \right)}^{2}}+\frac{\delta \eta^{3}{\left(a(km+r)(\beta +\delta -\rho )+kr(-\beta +\delta +\rho )\right)}^{3}}{a^{2}k^{3}{\left(1+r\eta \right)}^{3}{\left(\beta -\rho \right)}^{3}(-\beta +\delta +\rho )},+\frac{\eta {\left(-\beta +\delta +\rho \right)}^{2}(ar\delta +k(am\delta +r(-\beta +\delta +\rho )))}{a^{2}(k+kr\eta ){\left(\beta -\rho \right)}^{3}}-\frac{2\delta \eta^{2}(a(km+r)(\beta +\delta -\rho )+kr(-\beta +\delta +\rho ))(ar\delta +k(am\delta +r(-\beta +\delta +\rho )))}{a^{2}k^{2}{\left(1+r\eta \right)}^{2}{\left(\beta -\rho \right)}^{3}},\theta_{17}=\frac{\alpha \eta (k+2akm\eta +2ar\eta -kr\eta )}{k{\left(a+ar\eta \right)}^{2}}+\frac{\alpha \delta^{3}\eta (-k+a(km+r)\eta )(3ar\eta +k(3am\eta +2r\eta -1))}{a^{2}k^{2}{\left(1+r\eta \right)}^{3}{\left(-\beta +\rho \right)}^{3}}+\frac{\alpha \delta^{2}\eta \left(3k^{2}-4k(akm+(a+k)r)\eta -\left(k^{2}r^{2}+4akr(km+r)+6a^{2}{\left(km+r\right)}^{2}\right)\eta^{2}\right)}{a^{2}k^{2}{\left(1+r\eta \right)}^{3}{\left(\beta -\rho \right)}^{2}}+\frac{\alpha \delta \eta \left(-3k^{2}-2k(-kr+a(km+r))\eta +\left(2k^{2}r^{2}+4akr(km+r)-3a^{2}{\left(km+r\right)}^{2}\right)\eta^{2}\right)}{a^{2}k^{2}{\left(1+r\eta \right)}^{3}(\beta -\rho )},\theta_{18}=\frac{\alpha^{2}\eta^{2}(\beta -\delta -\rho )\left(\delta^{2}(-2+3am\eta +r\eta )+(1+r\eta )\rho^{2}+3\delta (\rho +am\eta \rho )-\beta (3\delta (1+am\eta ))\right)}{a^{2}{\left(1+r\eta \right)}^{3}{\left(\beta -\rho \right)}^{3}}+\frac{\alpha^{2}\eta^{2}\left(k\beta^{2}(1+r\eta )-3ar\delta \eta (\beta +\delta -\rho )\right)(\beta -\delta -\rho )}{a^{2}k{\left(1+r\eta \right)}^{3}{\left(\beta -\rho \right)}^{3}},\;\theta_{19}=-\frac{\alpha^{3}\delta \eta^{3}{\left(-\beta +\delta +\rho \right)}^{2}}{a^{2}{\left(1+r\eta \right)}^{3}{\left(\beta -\rho \right)}^{3}},$$

$$\theta_{21}=\frac{\eta (\beta +\rho )(ar\delta +k(am\delta +r(-\beta +\delta +\rho )))}{k\alpha (\beta +\beta \delta \eta -\rho )},\;\;\;\theta_{22}=1,\;\;\;\theta_{24}=\frac{\eta {\left(-\beta +\delta +\rho \right)}^{2}}{a(\beta +\beta \delta \eta -\rho )}\;\theta_{23}=\frac{\eta (\beta -\rho )(\beta -\delta -\rho )(1+\eta (\delta +\rho ))(ar\delta +k(am\delta +r(-\beta +\delta +\rho )))}{ak\alpha {\left(\beta +\beta \delta \eta -\rho \right)}^{2}},\theta_{25}=0,\;\;\;\theta_{26}=-\frac{\eta (\beta -\rho ){\left(-\beta +\delta +\rho \right)}^{2}{\left(1+\eta (\delta +\rho )\right)}^{2}(ar\delta +k(am\delta +r(-\beta +\delta +\rho )))}{a^{2}k\alpha {\left(\beta +\beta \delta \eta -\rho \right)}^{3}},\theta_{27}=\frac{\eta {\left(-\beta +\delta +\rho \right)}^{3}(1+\eta (\delta +\rho ))}{a^{2}{\left(\beta +\beta \delta \eta -\rho \right)}^{2}},\;\;\theta_{28}=0,\;\;\;\theta_{29}=0,\;\;\eta =\overset{ˆ}{\eta }+\eta_{1}.$$

The characteristic equation for the jacobian matrix of system (21) calculated at (0,0) is given as:

(22) $$\varrho^{2}-\overset{ˆ}{p}(\eta_{1})\varrho +\overset{ˆ}{q}(\eta_{1})=0,$$

where,

$$\overset{ˆ}{p}(\eta_{1})=2-\frac{\delta (\overset{ˆ}{\eta }+\eta_{1})(a(km+r)(\beta +\delta -\rho )-kr(\beta -\delta -\rho ))}{k(1+r(\overset{ˆ}{\eta }+\eta_{1}))(\beta -\rho )(\beta -\delta -\rho )},$$

$$\overset{ˆ}{q}(\eta_{1})=\frac{1}{1+r(\overset{ˆ}{\eta }+\eta_{1})}\left(1+r(\overset{ˆ}{\eta }+\eta_{1})+r\delta {\left(\overset{ˆ}{\eta }+\eta_{1}\right)}^{2}+\frac{\left(akm+(a+k)r)\delta (\overset{ˆ}{\eta }+\eta_{1}\right)}{k(\beta -\rho )}\right)-\frac{1}{1+r(\overset{ˆ}{\eta }+\eta_{1})}\left(\frac{\delta^{2}{\left(\overset{ˆ}{\eta }+\eta_{1}\right)}^{2}(a(km+r)+kr(1+\beta (\overset{ˆ}{\eta }+\eta_{1})))}{k(\beta +\beta \delta (\overset{ˆ}{\eta }+\eta_{1})-\rho )}-\frac{2a(km+r)\delta (\overset{ˆ}{\eta }+\eta_{1})}{k(\beta -\delta -\rho )}\right).$$

Consider that $(\alpha ,\beta ,\delta ,\rho ,\eta ,r,k,m)\in \Phi_{2}$. Then, the complex solutions for (22) are calculated as:

$$\varrho_{1}=\frac{\overset{ˆ}{p}(\eta_{1})-i\sqrt{4\overset{ˆ}{q}(\eta_{1})-{\overset{ˆ}{p}}^{2}(\eta_{1})}}{2}$$

and

$$\varrho_{1}=\frac{\overset{ˆ}{p}(\eta_{1})+i\sqrt{4\overset{ˆ}{q}(\eta_{1})-{\overset{ˆ}{p}}^{2}(\eta_{1}}}{2}.$$

Then, it follows that

$$|\varrho_{1}|=|\varrho_{2}|=\sqrt{2-\frac{\delta (\overset{ˆ}{\eta }+\eta_{1})(a(km+r)(\beta +\delta -\rho )-kr(\beta -\delta -\rho ))}{k(1+r(\overset{ˆ}{\eta }+\eta_{1}))(\beta -\rho )(\beta -\delta -\rho )}}.$$

Moreover, we get

(23) $${\left(\frac{d|\varrho_{1}|}{d\eta_{1}}\right)}_{\eta_{1}=0}=-\frac{\delta (a(km+r)(\beta +\delta -\rho )-kr(\beta -\delta -\rho ))}{2k{\left(1+r\overset{ˆ}{\eta }\right)}^{2}(\beta -\rho )(\beta -\delta -\rho )\sqrt{\overset{ˆ}{p}(0)}}\neq 0.$$

Since $|\overset{ˆ}{p}(0)|<2$ as $(\alpha ,\beta ,\delta ,\rho ,\overset{ˆ}{\eta },r,k,m)\in \Phi_{2}$. Moreover, a simple computation yields that

$$\overset{ˆ}{p}(0)=2-\frac{\delta \overset{ˆ}{\eta }(a(km+r)(\beta +\delta -\rho )-kr(\beta -\delta -\rho ))}{k(1+r\overset{ˆ}{\eta })(\beta -\rho )(\beta -\delta -\rho )},$$

and we suppose that $\overset{ˆ}{p}(0)\neq 0$ and $\overset{ˆ}{p}(0)\neq 1$, that is

(24) $$\overset{ˆ}{\eta }\neq -\frac{2k(\beta -\rho )(\beta -\delta -\rho )}{k(r(2\beta +\delta -2\rho )(\beta -\delta -\rho )-am\delta (\beta +\delta -\rho ))-ar\delta (\beta +\delta -\rho )},$$

and

(25) $$\overset{ˆ}{\eta }\neq \frac{k(\beta -\rho )(\beta -\delta -\rho )}{\left(\beta +\delta -\rho )(ar\delta +k(am\delta +r(-\beta +\delta +\rho ))\right)}.$$

Suppose that (24) and (25) holds and $(\alpha ,\beta ,\delta ,\rho ,\overset{ˆ}{\eta },r,k,m)\in \Phi_{2}$. Then it follows that $\overset{ˆ}{p}(0)\neq \pm 2,0,-1$, that is, $\varrho_{1}^{m},\varrho_{2}^{m}\neq 1$ for every m∈{1,2,3,4} about $\eta_{1}=0$. Consequently, both solutions of (22) does not lie inside the intersection of the unit disks with the coordinate axes when $\eta_{1}=0$. In the same way, we assume that $\lambda =\frac{\overset{ˆ}{p}(0)}{2}$, $\omega =\frac{1}{2}\sqrt{4\overset{ˆ}{q}(0)-{\overset{ˆ}{p}}^{2}(0)}$. Formerly, to change (21) into normal form, we take the following similarity transformation:

(26) $$\left(\begin{array}{c} x\\ y \end{array}\right)=\left(\begin{array}{cc} \theta_{12}\; & 0\\ \lambda -\theta_{11}\; & -\omega \end{array}\right)\left(\begin{array}{c} u\\ v \end{array}\right).$$

By using (26), we get the next typical form for (21):

(27) $$\left(\begin{array}{c} u\\ v \end{array}\right)\to \left(\begin{array}{cc} \lambda \; & -\omega \\ \omega \; & \lambda \end{array}\right)\left(\begin{array}{c} u\\ v \end{array}\right)+\left(\begin{array}{c} \overset{˜}{F}(u,v)\\ \overset{˜}{G}(u,v) \end{array}\right),$$

where,

$$\overset{˜}{F}(u,v)=u^{2}\theta_{12}\left(\theta_{13}+\left(u\lambda -v\omega -u\theta_{11}\right)\theta_{17}\right)+\frac{{\left(v\omega +u\theta_{11}-u\lambda \right)}^{2}\left(\theta_{15}+\left(u\lambda -v\omega -u\theta_{11}\right)\theta_{19}\right)}{\theta_{12}}+u\left(u\lambda -v\omega -u\theta_{11}\right)\left(\theta_{14}+\left(u\lambda -v\omega -u\theta_{11}\right)\theta_{18}\right)+u^{3}\theta_{12}^{2}\theta_{16}+O\left({\left(|u|+|v|\right)}^{4}\right),$$

$$\overset{˜}{G}(u,v)=u^{2}\theta_{12}^{2}\left(\frac{\left(\lambda -\theta_{11}\right)\theta_{13}}{\omega \theta_{12}}-\frac{\theta_{23}}{\omega }\right)-u\left(v\omega -u\left(\lambda -\theta_{11}\right)\right)\theta_{12}\left(\frac{\left(\lambda -\theta_{11}\right)\theta_{14}}{\omega \theta_{12}}-\frac{\theta_{24}}{\omega }\right)+\left(u\left(\lambda -\theta_{11}\right)-v\omega \right){}^{2}\left(\frac{\left(\lambda -\theta_{11}\right)\theta_{15}}{\omega \theta_{12}}-\frac{\theta_{25}}{\omega }\right)+u^{3}\theta_{12}^{3}\left(\frac{\left(\lambda -\theta_{11}\right)\theta_{16}}{\omega \theta_{12}}-\frac{\theta_{26}}{\omega }\right)+u^{2}\left(u\left(\lambda -\theta_{11}\right)-v\omega \right)\theta_{12}^{2}\left(\frac{\left(\lambda -\theta_{11}\right)\theta_{17}}{\omega \theta_{12}}\right)+u^{2}\left(u\left(\lambda -\theta_{11}\right)-v\omega \right)\theta_{12}^{2}\left(\frac{\theta_{27}}{\omega }\right)+u\left(u\left(\lambda -\theta_{11}-v\omega \right)\right){}^{2}\theta_{12}\left(\frac{\left(\lambda -\theta_{11}\right)\theta_{18}}{\omega \theta_{12}}-\frac{\theta_{28}}{\omega }\right)+\left(u\left(\lambda -\theta_{11}\right)-v\omega \right){}^{3}\left(\frac{\left(\lambda -\theta_{11}\right)\theta_{19}}{\omega \theta_{12}}-\frac{\theta_{29}}{\omega }\right)+O\left({\left(|u|+|v|\right)}^{4}\right).$$

Therefore, we have the following non-zero real numbers:

$$Ϝ={\left(\left[-Re\left(\frac{(1-2\varrho_{1})\varrho_{2}^{2}}{1-\varrho_{1}}\xi_{20}\xi_{11}\right)-\frac{1}{2}|\xi_{11}{|}^{2}-|\xi_{02}{|}^{2}+Re(\varrho_{2}\xi_{21})\right]\right)}_{\eta_{1}=0},$$

where,

$$\xi_{20}=\frac{i\left(\left(\lambda -i\omega -\theta_{11}\right){\left(\lambda +i\omega -\theta_{11}\right)}^{2}\theta_{15}-\theta_{12}^{3}\theta_{23}+\theta_{12}^{2}\left(\left(\lambda -i\omega -\theta_{11}\right)\theta_{13}+\left(-\lambda -i\omega +\theta_{11}\right)\theta_{24}\right)\right)}{4\omega \theta_{12}}+\frac{i\left(\left(\lambda^{2}+\omega^{2}-2\lambda \theta_{11}+\theta_{11}^{2}\right)\theta_{14}-{\left(\lambda +i\omega -\theta_{11}\right)}^{2}\theta_{25}\right)}{4\omega },\;\;\xi_{11}=\frac{i\left(\lambda +i\omega -\theta_{11}\right){\left(-\lambda +i\omega +\theta_{11}\right)}^{2}\theta_{15}}{2\omega \theta_{12}}-\frac{i\theta_{12}\left(\left(-\lambda +i\omega +\theta_{11}\right)\theta_{13}+\theta_{12}\theta_{23}+\left(\lambda -\theta_{11}\right)\theta_{24}\right)}{2\omega }+\frac{\left(i\lambda +\omega -i\theta_{11}\right)\left(\left(\lambda -\theta_{11}\right)\theta_{14}+\left(-\lambda -i\omega +\theta_{11}\right)\theta_{25}\right)}{2\omega },\;\;\xi_{02}=\frac{i\left({\left(\lambda -i\omega -\theta_{11}\right)}^{3}\theta_{15}-\theta_{12}^{3}\theta_{23}\right)}{4\omega \theta_{12}}+\frac{i\left(\left(\lambda -i\omega -\theta_{11}\right)\theta_{12}\left(\theta_{13}-\theta_{24}\right)-{\left(\lambda -i\omega -\theta_{11}\right)}^{2}\left(\theta_{14}-\theta_{25}\right)\right)}{4\omega },\xi_{21}=\frac{i\left(3{\left(\lambda^{2}+\omega^{2}-2\lambda \theta_{11}+\theta_{11}^{2}\right)}^{2}\theta_{19}-3\theta_{12}^{4}\theta_{26}+\theta_{12}^{3}\left(3\left(\lambda -i\omega -\theta_{11}\right)\theta_{16}-\left(3\lambda +i\omega -3\theta_{11}\right)\theta_{27}\right)\right)}{8\omega \theta_{12}}+\frac{\theta_{12}\left(\left(3\lambda +i\omega -3\theta_{11}\right)\left(i\lambda +\omega -i\theta_{11}\right)\theta_{17}-\left(3\lambda -i\omega -3\theta_{11}\right)\left(i\lambda -\omega -i\theta_{11}\right)\theta_{28}\right)}{8\omega }+\frac{\left(\lambda^{2}+\omega^{2}-2\lambda \theta_{11}+\theta_{11}^{2}\right)\left(\left(3i\lambda +\omega -3i\theta_{11}\right)\theta_{18}-3\left(i\lambda -\omega -i\theta_{11}\right)\theta_{29}\right)}{8\omega }.$$

Hence, we get the following theorem about the existence of Neimark-Sacker bifurcation at the fixed point $\bar{x}$ of the system (4) (see [40], [41], [42], [43], [44]).

Theorem 4.1 *Assume that*(24)*holds true and*Ϝ≠0*. Then, the positive fixed point*

$$\bar{x}=\left(\frac{a\delta }{\beta -\delta -\rho },\frac{a(\beta -\rho )((kr(\beta -\delta -\rho )-akm\delta )-ar\delta )}{k\alpha {\left(-\beta +\delta +\rho \right)}^{2}}\right)$$

*of system*(3)*experiences the Neimark-Sacker bifurcation, whenever η changes in least neighbourhood of*

$$\overset{ˆ}{\eta }=\frac{2}{-\beta +\delta +\rho }-\frac{akm+(a+k)r}{ar\delta +k(am\delta +r(-\beta +\delta +\rho ))}.$$

*In addition, if*Ϝ<0,(Ϝ>0)*, respectively, then an attracting or repelling invariant closed curve bifurcates from the equilibrium point for*$\eta >\overset{ˆ}{\eta }(\eta <\overset{ˆ}{\eta })$*, respectively.*

## 5 Period-doubling bifurcation

This section is centred on the existence of period-doubling bifurcation about the positive equilibrium point of the system (3). For the study of this type of bifurcation, a centre manifold theorem is applied after using the normal forms to display the presence and direction of such kind of bifurcation. Freshly, various scientists have studied the period-doubling bifurcation related to discrete-time mathematical models [41], [42], [43], [44]. One can see that $\psi_{1}=-1$ and $|\psi_{2}|\neq 1$ if and only if

$$a=-\frac{k(-\beta +\delta +\rho )\left(-\beta \left(4+r\delta \eta^{2}\right)+4\rho +r\delta \eta (-2+\eta (\delta +\rho ))\right)}{\left(km+r)\delta \eta (-\beta (2+\delta \eta )+2\rho +\delta (-2+\eta (\delta +\rho ))\right)}.$$

Furthermore, under the supposition that β>δ+ρ and kr(β−δ−ρ)<aδ(km−r), we study the following set:

$$\Phi_{1}=\{\alpha ,\beta ,\delta ,\rho ,\eta ,r,k,m\in {ℜ}^{+}:a=-\frac{k(-\beta +\delta +\rho )\left(-\beta \left(4+r\delta \eta^{2}\right)+4\rho +r\delta \eta (-2+\eta (\delta +\rho ))\right)}{\left(km+r)\delta \eta (-\beta (2+\delta \eta )+2\rho +\delta (-2+\eta (\delta +\rho ))\right)}\}.$$

Then, by taking *η* as the parameter for bifurcation, the positive equilibrium point $\bar{x}$ of the system (3) experiences the period-doubling bifurcation. Moreover, the bifurcation parameter *η* ranges in a small neighbourhood of $\overset{ˆ}{\eta }$, which is obtained from:

$$a=-\frac{k(-\beta +\delta +\rho )\left(-\beta \left(4+r\delta {\overset{ˆ}{\eta }}^{2}\right)+4\rho +r\delta \overset{ˆ}{\eta }(-2+\overset{ˆ}{\eta }(\delta +\rho ))\right)}{\left(km+r)\delta \overset{ˆ}{\eta }(-\beta (2+\delta \overset{ˆ}{\eta })+2\rho +\delta (-2+\overset{ˆ}{\eta }(\delta +\rho ))\right)}.$$

Additionally, the mathematical system (3) is categorized uniformly with the following planer map:

(28) $$\left(\begin{array}{c} p\\ z \end{array}\right)\to \left(\begin{array}{c} p+\eta \left(r(1-\frac{p}{k})-\frac{\alpha pz}{a+p}-mp^{2}\right)\\ z+\eta \left(\frac{\beta pz}{a+p}-\delta z-\frac{\rho pz}{a+p}\right) \end{array}\right).$$

To examine the period-doubling bifurcation about the one and only fixed point

$$\bar{x}=\left(\frac{a\delta }{\beta -\delta -\rho },\frac{a(\beta -\rho )((kr(\beta -\delta -\rho )-akm\delta )-ar\delta )}{k\alpha {\left(-\beta +\delta +\rho \right)}^{2}}\right)$$

of (28), we suppose that $(\alpha ,\beta ,\delta ,\rho ,\overset{ˆ}{\eta },r,k,m)\in \Phi_{1}$. Then it follows that

(29) $$\left(\begin{array}{c} p\\ z \end{array}\right)\to \left(\begin{array}{c} p+\overset{ˆ}{\eta }\left(r(1-\frac{p}{k})-\frac{\alpha pz}{a+p}-mp^{2}\right)\\ z+\overset{ˆ}{\eta }\left(\frac{\beta pz}{a+p}-\delta z-\frac{\rho pz}{a+p}\right) \end{array}\right).$$

We take $\overset{¯}{\eta }$ as a small bifurcation parameter, then the perturbation of mapping (28) can be defined by the following map:

(30) $$\left(\begin{array}{c} p\\ z \end{array}\right)\to \left(\begin{array}{c} p+(\overset{ˆ}{\eta }+\overset{¯}{\eta })\left(r(1-\frac{p}{k})-\frac{\alpha pz}{a+p}-mp^{2}\right)\\ z+(\overset{ˆ}{\eta }+\overset{¯}{\eta })\left(\frac{\beta pz}{a+p}-\delta z-\frac{\rho pz}{a+p}\right) \end{array}\right),$$

where $|\overset{¯}{\eta }|\ll 1$, is a small parameter for perturbation. Taking $x=p-\frac{a\delta }{\beta -\delta -\rho }$ and $y=z-\frac{a(\beta -\rho )((kr(\beta -\delta -\rho )-akm\delta )-ar\delta )}{k\alpha {\left(-\beta +\delta +\rho \right)}^{2}}$. Then, from (30) we have the following map whose equilibrium point is at (0,0);

(31) $$\left(\begin{array}{c} x\\ y \end{array}\right)\to \left(\begin{array}{cc} \theta_{11}\; & \theta_{12}\\ \theta_{21}\; & \theta_{22} \end{array}\right)\left(\begin{array}{c} x\\ y \end{array}\right)+\left(\begin{array}{c} f_{1}(x,y,\overset{¯}{\eta })\\ f_{2}(x,y,\overset{¯}{\eta }) \end{array}\right),$$

with

$$f_{1}(x,y,\overset{¯}{\eta })=\theta_{13}x^{2}+\theta_{14}xy+\theta_{15}x\overset{¯}{\eta }+\theta_{16}y\overset{¯}{\eta }+\theta_{17}x^{3}+\theta_{18}x^{2}y+\theta_{19}\overset{¯}{\eta }x^{2}+\theta_{20}xy\overset{¯}{\eta }+O\left({\left(|x|+|y|+|\overset{¯}{\eta }|\right)}^{4}\right),f_{2}(x,y,\overset{¯}{\eta })=\theta_{23}x^{2}+\theta_{24}xy+\theta_{25}x\overset{¯}{\eta }+\theta_{26}y\overset{¯}{\eta }+\theta_{27}x^{3}+\theta_{28}x^{2}y+\theta_{29}\overset{¯}{\eta }x^{2}+\theta_{30}xy\overset{¯}{\eta }+O\left({\left(|x|+|y|+|\overset{¯}{\eta }|\right)}^{4}\right),\theta_{11}=\frac{k{\left(a+p\right)}^{2}-p\left({\left(a+p\right)}^{2}(2km+r)-kz\alpha \right)\overset{¯}{\eta }}{k{\left(a+p\right)}^{2}},\;\theta_{12}=-\frac{p\alpha \overset{¯}{\eta }}{a+p},\theta_{13}=-\frac{\left(m{\left(a+p\right)}^{3}+pz\alpha \right)\overset{¯}{\eta }}{{\left(a+p\right)}^{3}},\;\theta_{14}=\frac{p\alpha \overset{¯}{\eta }}{{\left(a+p\right)}^{2}},\;\theta_{15}=p\left(-2m-\frac{r}{k}+\frac{z\alpha }{{\left(a+p\right)}^{2}}\right),\theta_{16}=-\frac{p\alpha }{a+p},\;\theta_{17}=\frac{pz\alpha \overset{¯}{\eta }}{{\left(a+p\right)}^{4}},\;\theta_{18}=-\frac{p\alpha \overset{¯}{\eta }}{{\left(a+p\right)}^{3}},\;\theta_{19}=-m-\frac{pz\alpha }{{\left(a+p\right)}^{3}},\;\theta_{20}=\frac{p\alpha }{{\left(a+p\right)}^{2}},\theta_{21}=\frac{az\overset{¯}{\eta }(\beta -\rho )}{{\left(a+p\right)}^{2}},\;\theta_{22}=-\frac{\overset{¯}{\eta }(a\delta +p(-\beta +\delta +\rho ))}{a+p},\;\theta_{23}=\frac{az\overset{¯}{\eta }(\rho -\beta )}{{\left(a+p\right)}^{3}},\;\theta_{24}=\frac{az(\beta -\rho )}{{\left(a+p\right)}^{2}},\theta_{25}=\frac{a\overset{¯}{\eta }(\beta -\rho )}{{\left(a+p\right)}^{2}},\;\theta_{26}=-\frac{a\delta +p(-\beta +\delta +\rho )}{a+p},\;\theta_{27}=\frac{az\overset{¯}{\eta }(\beta -\rho )}{{\left(a+p\right)}^{4}},\;\theta_{28}=-\frac{a\overset{¯}{\eta }(\beta -\rho )}{{\left(a+p\right)}^{3}},\theta_{29}=-\frac{az(\beta -\rho )}{{\left(a+p\right)}^{3}},\;\theta_{30}=\frac{a(\beta -\rho )}{{\left(a+p\right)}^{2}}.$$

Taking $(\alpha ,\beta ,\delta ,\rho ,\overset{ˆ}{\eta },r,k,m)\in \Phi_{1}$, the roots of (12) are computed as

$$\psi_{1}=-1\;\;\text{and}\;\psi_{2}=3+\frac{(akm+(a+k)r)\delta \overset{¯}{\eta }}{k(\beta -\rho )}+\frac{2a(km+r)\delta \overset{¯}{\eta }}{k(-\beta +\delta +\rho )}.$$

Afterwards, one has the next translation;

(32) $$\left(\begin{array}{c} x\\ y \end{array}\right)=S\left(\begin{array}{c} u\\ v \end{array}\right),$$

where,

$$S=\left[\begin{array}{cc} -\frac{p\alpha \overset{¯}{\eta }}{a+p}\; & -\frac{p\alpha \overset{¯}{\eta }}{a+p}\\ \frac{-2k{\left(a+p\right)}^{2}+p\left({\left(a+p\right)}^{2}(2km+r)-kz\alpha \right)\overset{¯}{\eta }}{k{\left(a+p\right)}^{2}}\; & 3+\frac{-k{\left(a+p\right)}^{2}+p\left({\left(a+p\right)}^{2}(2km+r)-kz\alpha \right)\overset{¯}{\eta }}{k{\left(a+p\right)}^{2}}+\frac{(akm+(a+k)r)\delta \overset{¯}{\eta }}{k(\beta -\rho )}+\frac{2a(km+r)\delta \overset{¯}{\eta }}{k(-\beta +\delta +\rho )} \end{array}\right]$$

be a nonsingular matrix. By applying transformation (32), the map (31) can be written as:

(33) $$\left(\begin{array}{c} u\\ v \end{array}\right)\to \left(\begin{array}{cc} -1\; & 0\\ 0\; & 3+\frac{(akm+(a+k)r)\delta \overset{¯}{\eta }}{k(\beta -\rho )}+\frac{2a(km+r)\delta \overset{¯}{\eta }}{k(-\beta +\delta +\rho )} \end{array}\right)\left(\begin{array}{c} u\\ v \end{array}\right)+\left(\begin{array}{c} f(u,v,\overset{¯}{\eta })\\ g(u,v,\overset{¯}{\eta }) \end{array}\right),$$

where,

$$f(u,v,\overset{¯}{\eta })=\left(\frac{\left(\psi_{2}-\theta_{11}\right)\theta_{19}}{\theta_{12}\left(\psi_{2}+1\right)}-\frac{\theta_{29}}{\psi_{2}+1}\right)\overset{¯}{\eta }\;x^{2}+\left(\frac{\left(\psi_{2}-\theta_{11}\right)\theta_{20}}{\theta_{12}\left(\psi_{2}+1\right)}-\frac{\theta_{30}}{\psi_{2}+1}\right)\overset{¯}{\eta }\;xy+\left(\frac{\left(\psi_{2}-\theta_{11}\right)\theta_{15}}{\theta_{12}\left(\psi_{2}+1\right)}-\frac{\theta_{25}}{\psi_{2}+1}\right)\overset{¯}{\eta }\;x+\left(\frac{\left(\psi_{2}-\theta_{11}\right)\theta_{16}}{\theta_{12}\left(\psi_{2}+1\right)}-\frac{\theta_{26}}{\psi_{2}+1}\right)y\overset{¯}{\eta }+\left(\frac{\left(\psi_{2}-\theta_{11}\right)\theta_{17}}{\theta_{12}\left(\psi_{2}+1\right)}-\frac{\theta_{27}}{\psi_{2}+1}\right)x^{3}+\left(\frac{\left(\psi_{2}-\theta_{11}\right)\theta_{18}}{\theta_{12}\left(\psi_{2}+1\right)}-\frac{\theta_{28}}{\psi_{2}+1}\right)x^{2}y+\left(\frac{\left(\psi_{2}-\theta_{11}\right)\theta_{13}}{\theta_{12}\left(\psi_{2}+1\right)}-\frac{\theta_{23}}{\psi_{2}+1}\right)x^{2}+\left(\frac{\left(\psi_{2}-\theta_{11}\right)\theta_{14}}{\theta_{12}\left(\psi_{2}+1\right)}-\frac{\theta_{24}}{\psi_{2}+1}\right)yx+O\left({\left(|u|+|v|+|\overset{¯}{\eta }|\right)}^{4}\right),$$

$$g(u,v,\overset{¯}{\eta })=\left(\frac{\left(1+\theta_{11}\right)\theta_{19}}{\theta_{12}\left(\psi_{2}+1\right)}+\frac{\theta_{29}}{\psi_{2}+1}\right)\overset{¯}{\eta }\;x^{2}+\left(\frac{\left(1+\theta_{11}\right)\theta_{20}}{\theta_{12}\left(\psi_{2}+1\right)}+\frac{\theta_{30}}{\psi_{2}+1}\right)\overset{¯}{\eta }\;xy+\left(\frac{\left(1+\theta_{11}\right)\theta_{15}}{\theta_{12}\left(\psi_{2}+1\right)}+\frac{\theta_{25}}{\psi_{2}+1}\right)\overset{¯}{\eta }\;x+\left(\frac{\left(1+\theta_{11}\right)\theta_{16}}{\theta_{12}\left(\psi_{2}+1\right)}+\frac{\theta_{26}}{\psi_{2}+1}\right)y\overset{¯}{\eta }+\left(\frac{\left(1+\theta_{11}\right)\theta_{17}}{\theta_{12}\left(\psi_{2}+1\right)}+\frac{\theta_{27}}{\psi_{2}+1}\right)x^{3}+\left(\frac{\left(1+\theta_{11}\right)\theta_{18}}{\theta_{12}\left(\psi_{2}+1\right)}+\frac{\theta_{28}}{\psi_{2}+1}\right)x^{2}y+\left(\frac{\left(1+\theta_{11}\right)\theta_{13}}{\theta_{12}\left(\psi_{2}+1\right)}+\frac{\theta_{23}}{\psi_{2}+1}\right)x^{2}+\left(\frac{\left(1+\theta_{11}\right)\theta_{14}}{\theta_{12}\left(\psi_{2}+1\right)}+\frac{\theta_{24}}{\psi_{2}+1}\right)yx+O\left({\left(|u|+|v|+|\overset{¯}{\eta }|\right)}^{4}\right),$$

where,

$$x=\theta_{12}(u+v),\;\;\;\;\;\;\;\;\;y=-(1+\theta_{11})u+(\psi_{2}-\theta_{11})v.$$

Assume that ${℧}^{c}(0,0,0)$ be the centre manifold of (33) designed at (0,0) in a least neighbourhood of $\overset{¯}{\eta }=0$. Then ${℧}^{c}(0,0,0)$ can be approximated as:

$${℧}^{c}(0,0,0)=\left\{(u,v,\overset{¯}{\eta })\in R^{3}:v=M_{11}u^{2}+M_{12}u\overset{¯}{\eta }+M_{13}{\overset{¯}{\eta }}^{2}+O\left({\left(|\overset{¯}{\eta }|+|u|\right)}^{3}\right)\right\},$$

where,

$$M_{11}=\frac{1}{1-\psi_{2}}\left(\left(\frac{\left(1+\theta_{11}\right)\theta_{13}}{\theta_{12}\left(\psi_{2}+1\right)}+\frac{\theta_{23}}{\psi_{2}+1}\right){\theta_{12}}^{2}-\left(\frac{\left(1+\theta_{11}\right)\theta_{14}}{\theta_{12}\left(\psi_{2}+1\right)}+\frac{\theta_{24}}{\xi_{2}+1}\right)\left(1+\theta_{11}\right)\theta_{12}\right),$$

$$M_{12}=\frac{1}{1-\psi_{2}}\left(\left(\frac{\left(1+\theta_{11}\right)\theta_{15}}{\theta_{12}\left(\psi_{2}+1\right)}+\frac{\theta_{25}}{\psi_{2}+1}\right)\theta_{12}-\left(\frac{\left(1+\theta_{11}\right)\theta_{16}}{\theta_{12}\left(\psi_{2}+1\right)}+\frac{\theta_{26}}{\psi_{2}+1}\right)\left(1+\theta_{11}\right)\right),$$

$$M_{13}=0.$$

Hence, the mapping centred to set ${℧}^{c}(0,0,0)$ is defined in such a way:

$$G:u\to -u+a_{11}u^{2}+a_{12}u\overset{¯}{\eta }+a_{13}u^{2}\overset{¯}{\eta }+a_{14}u{\overset{¯}{\eta }}^{2}+a_{15}u^{3}+O\left({\left(|u|+|\overset{¯}{\eta }|\right)}^{4}\right),$$

where,

$$a_{11}=\frac{\theta_{11}^{2}\theta_{14}-\theta_{12}^{2}\theta_{23}+\theta_{11}\left(\theta_{12}\left(\theta_{24}-\theta_{13}\right)-\theta_{14}\left(\xi_{2}-1\right)\right)-\theta_{14}\xi_{2}+\theta_{12}\left(\theta_{24}+\theta_{13}\psi_{2}\right)}{1+\psi_{2}},$$

$$a_{12}=\frac{\theta_{11}^{2}\theta_{16}-\theta_{12}^{2}\theta_{25}+\theta_{11}\left(\theta_{12}\left(\theta_{26}-\theta_{15}\right)-\theta_{16}\left(\psi_{2}-1\right)\right)-\theta_{16}\psi_{2}+\theta_{12}\left(\theta_{26}+\theta_{15}\psi_{2}\right)}{\theta_{12}\left(1+\psi_{2}\right)},$$

$$a_{13}=\left(\frac{\left(\theta_{11}-\psi_{2}\right)\theta_{20}}{\theta_{12}\left(\psi_{2}+1\right)}+\frac{\theta_{30}}{\psi_{2}+1}\right)\theta_{12}\left(1+\theta_{11}\right)-\left(\frac{\left(\theta_{11}-\psi_{2}\right)\theta_{19}}{\theta_{12}\left(\psi_{2}+1\right)}+\frac{\theta_{29}}{\psi_{2}+1}\right){\theta_{12}}^{2}-\left(\frac{\left(\theta_{11}-\psi_{2}\right)\theta_{15}}{\theta_{12}\left(\psi_{2}+1\right)}+\frac{\theta_{25}}{\psi_{2}+1}\right)\theta_{12}M_{1}-\left(\frac{\left(\theta_{11}-\psi_{2}\right)\theta_{16}}{\theta_{12}\left(\psi_{2}+1\right)}+\frac{\theta_{26}}{\psi_{2}+1}\right)\left(\psi_{2}-\theta_{11}\right)M_{1}-2\;\left(\frac{\left(\theta_{11}-\psi_{2}\right)\theta_{13}}{\theta_{12}\left(\psi_{2}+1\right)}+\frac{\theta_{23}}{\psi_{2}+1}\right){\theta_{12}}^{2}M_{2}+\left(\frac{\left(\theta_{11}-\psi_{2}\right)\theta_{14}}{\theta_{12}\left(\psi_{2}+1\right)}+\frac{\theta_{24}}{\psi_{2}+1}\right)\left(1+\theta_{11}\right)\theta_{12}M_{2}-\left(\frac{\left(\theta_{11}-\psi_{2}\right)\theta_{14}}{\theta_{12}\left(\psi_{2}+1\right)}+\frac{\theta_{24}}{\psi_{2}+1}\right)\left(\psi_{2}-\theta_{11}\right)M_{2}\theta_{12},$$

$$a_{14}=\left(\frac{\left(\theta_{11}-\psi_{2}\right)\theta_{14}}{\theta_{12}\left(\psi_{2}+1\right)}+\frac{\theta_{24}}{\psi_{2}+1}\right)\left(1+\theta_{11}\right)\theta_{12}M_{3}-\left(\frac{\left(\theta_{11}-\psi_{2}\right)\theta_{15}}{\theta_{12}\left(\psi_{2}+1\right)}+\frac{\theta_{25}}{\psi_{2}+1}\right)\theta_{12}M_{2}-\left(\frac{\left(\theta_{11}-\psi_{2}\right)\theta_{16}}{\theta_{12}\left(\psi_{2}+1\right)}+\frac{\theta_{26}}{\psi_{2}+1}\right)\left(\psi_{2}-\theta_{11}\right)M_{2}-2\;\left(\frac{\left(\theta_{11}-\psi_{2}\right)\theta_{13}}{\theta_{12}\left(\psi_{2}+1\right)}+\frac{\theta_{23}}{\psi_{2}+1}\right){\theta_{12}}^{2}M_{3}-\left(\frac{\left(\theta_{11}-\psi_{2}\right)\theta_{14}}{\theta_{12}\left(\psi_{2}+1\right)}+\frac{\theta_{24}}{\psi_{2}+1}\right)\left(\psi_{2}-\theta_{11}\right)M_{3}\theta_{12},$$

$$a_{15}=\frac{M_{3}\left(\theta_{11}^{2}\theta_{16}-\theta_{12}^{2}\theta_{25}+\theta_{12}\left(\theta_{15}-\theta_{26}\right)\psi_{2}+\theta_{16}\psi_{2}^{2}+\theta_{11}\left(\theta_{12}\left(-\theta_{15}+\theta_{26}\right)-2\theta_{16}\psi_{2}\right)\right)}{\theta_{12}\left(1+\psi_{2}\right)}=0.$$

Next, we have the following real numbers:

$$L_{11}={\left(\frac{\partial^{2}f_{1}}{\partial u\partial \overset{¯}{\eta }}+\frac{1}{2}\frac{\partial G}{\partial \overset{¯}{\eta }}\frac{\partial^{2}G}{\partial u^{2}}\right)}_{\left(0,0\right)}=\frac{\theta_{11}^{2}\theta_{16}-\theta_{12}^{2}\theta_{25}+\theta_{11}\left(\theta_{12}\left(\theta_{26}-\theta_{15}\right)-\theta_{16}\left(\psi_{2}-1\right)\right)-\theta_{16}\psi_{2}+\theta_{12}\left(\theta_{26}+\theta_{15}\psi_{2}\right)}{\theta_{12}\left(1+\psi_{2}\right)}\neq 0,$$

$$L_{12}={\left({\left(\frac{1}{2}\frac{\partial^{2}G}{\partial u^{2}}\right)}^{2}+\frac{1}{6}\frac{\partial^{3}G}{\partial u^{3}}\right)}_{\left(0,0\right)}=\frac{{\left(\theta_{12}^{2}\theta_{23}-\theta_{11}^{2}\theta_{14}+\theta_{11}\left(\theta_{12}\left(\theta_{13}-\theta_{24}\right)+\theta_{14}\left(\psi_{2}-1\right)\right)+\theta_{14}\psi_{2}-\theta_{12}\left(\theta_{24}+\theta_{13}\psi_{2}\right)\right)}^{2}}{{\left(1+\psi_{2}\right)}^{2}}\neq 0.$$

Hence, by the study as mentioned earlier, we have the following result connected to the presence and direction of period-doubling bifurcation for mathematical system (3) about $\overset{¯}{x}$.

Theorem 5.1 *Assume that the parameter η changes in least neighbourhood of*$\overset{¯}{\eta }$*and*$L_{12}\neq 0$*, then the system*(3)*experiences the period-doubling bifurcation at one and only positive equilibrium*$\overset{¯}{x}$*. Additionally, the period-two trajectories that bifurcates from*$\overset{¯}{x}$*are stable for*$L_{12}>0$*, and if*$L_{12}<0$*, then these trajectories are unstable.*

## 6 Chaos control

To control inconsistent, accidental and irregular behaviour in any biological system, chaos control is well thought-out to be a practical tool for avoiding this complex and disordered behaviour. For additional details associated with the biological significance of chaos control and its applied use in the real world, we mention to [45]. In this part of the manuscript, we use a simple chaos control method for the system (3). Furthermore, there are many chaos control techniques for discrete dynamical systems. We refer a reader to [43], [44], [45], [46], [47], [48], [49] for additional details connected to these methods. We implement a generalized hybrid control technique (see [33], [34], [35], [36], [37]). The generalized hybrid control method [48] is centred on parameter perturbation and a state feedback control technique. By implementing generalized hybrid control methodology (with control parameter Θ∈(0,1]) to the system (3), we get:

(34) $$p_{n+1}=\Theta^{3}\left(p_{n}+\eta \left(rp_{n}(1-\frac{p_{n}}{k})-\frac{\alpha p_{n}z_{n}}{a+p_{n}}-mp_{n}^{2}\right)\right)+(1-\Theta^{3})p_{n},z_{n+1}=\Theta^{3}\left(z_{n}+\eta \left(\frac{\beta p_{n}z_{n}}{a+p_{n}}-\delta z_{n}-\frac{\rho p_{n}z_{n}}{a+p_{n}}\right)\right)+(1-\Theta^{3})z_{n}.$$

Then, system (34) is controllable provided that its fixed point

$$\bar{x}=\left(\frac{a\delta }{\beta -\delta -\rho },\frac{a(\beta -\rho )((kr(\beta -\delta -\rho )-akm\delta )-ar\delta )}{k\alpha {\left(-\beta +\delta +\rho \right)}^{2}}\right),$$

is locally asymptotically stable. Additionally, the jacobian matrix for system (34) at its positive fixed point $\bar{x}$ is calculated as follows:

$$\left[\begin{array}{cc} 1+\frac{(akm+(a+k)r)\delta \eta \Theta^{3}}{k(\beta -\rho )}+\frac{2a(km+r)\delta \eta \Theta^{3}}{k(-\beta +\delta +\rho )}\; & \frac{\alpha \delta \eta \Theta^{3}}{-\beta +\rho }\\ -\frac{\eta \Theta^{3}(ar\delta +k(am\delta +r(-\beta +\delta +\rho )))}{k\alpha }\; & 1 \end{array}\right].$$

Formerly, the characteristic polynomial for the above-mentioned jacobian matrix is specified by

$$G(\lambda )=\lambda^{2}-\lambda \left(2+\frac{(akm+(a+k)r)\delta \eta \Theta^{3}}{k(\beta -\rho )}+\frac{2a(km+r)\delta \eta \Theta^{3}}{k(-\beta +\delta +\rho )}\right)+1+r\delta \eta^{2}\Theta^{6}+\frac{(akm+(a+k)r)\delta \eta \Theta^{3}\left(-1+\delta \eta \Theta^{3}\right)}{k(-\beta +\rho )}+\frac{2a(km+r)\delta \eta \Theta^{3}}{k(-\beta +\delta +\rho )}.$$

Lemma 6.1 *Assume that*β>δ+ρ*, and*kr(β−δ−ρ)<aδ(km−r)*. Furthermore, let*$\bar{x}$*be unique positive fixed point of system*(34)*, then*$\bar{x}$*is locally asymptotically stable if and only if for*

$$r\eta \Theta^{3}+\frac{(akm+(a+k)r)\left(-1+\delta \eta \Theta^{3}\right)}{k(-\beta +\rho )}+\frac{2a(km+r)}{k(-\beta +\delta +\rho )}<0,$$

*we have*

$$4+am\delta \eta \Theta^{3}\left(\frac{-2+\delta \eta \Theta^{3}}{-\beta +\rho }+\frac{4}{-\beta +\delta +\rho }\right)+r\delta \eta \Theta^{3}\left(\eta \Theta^{3}+\frac{(a+k)\left(-2+\delta \eta \Theta^{3}\right)}{k(-\beta +\rho )}+\frac{4a}{k(-\beta +\delta +\rho )}\right)>0.$$

## 7 Numerical simulations

**Figure 3:**
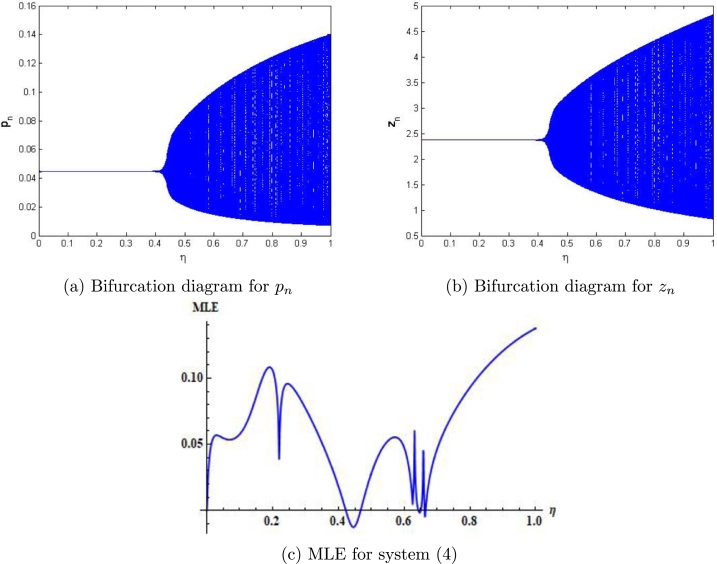
Plots of system (4) for *r* = 2.4917,*m* = 0.217,*β* = 2.09,*δ* = 0.32,*a* = 0.217,*k* = 0.217,*α* = 0.217,*ρ* = 0.217, with *p*_0_ = 0.0442580082377936,*z*_0_ = 2.3740415103508146 and *η* ∈ (0,1].

**Figure 4:**
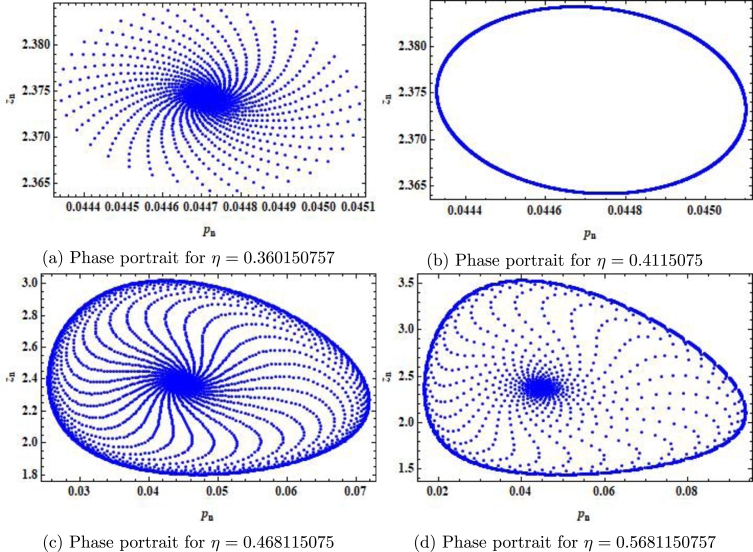
Phase portraits of system (4) for *r* = 2.4917,*m* = 0.217,*β* = 2.09,*δ* = 0.32,*a* = 0.217,*k* = 0.217,*α* = 0.217,*ρ* = 0.217, with *p*_0_ = 0.0442580082377936,*z*_0_ = 2.3740415103508146 and *η* ∈ (0,1].

Example 7.1 We choose r=2.4917,m=0.217,β=2.09,δ=0.32,a=0.217,k=0.217,α=0.217,ρ=0.217, $p_{0}=0.0442580082377936,z_{0}=2.3740415103508146$ and η∈(0,1]. Then, the mathematical system (4) experiences the Neimark-Sacker bifurcation whenever the bifurcation parameter *η* certainly passes through η=0.4115075. Furthermore, for these values, bifurcation diagrams and MLE are shown in Fig. 3 (a)-(c). In addition, some phase portraits for system (4) are shown in Fig. 4 (a)-(d), for η=0.368115075, 0.4118115075,0.468115075, and η=0.568115075, respectively.

Example 7.2 We choose a=0.8559,r=3.0609,β=4.8959,k=1.258,α=3.499,ρ=2.9,δ=0.47122,m=0.9, $p_{0}=0.26452580082377936,z_{0}=0.7740415103508146$ and η∈(0,1]. Then, the mathematical system (3) experiences the Neimark-Sacker bifurcation whenever the bifurcation parameter *η* certainly passes through η=0.4681150757612096. Furthermore, for these values the jacobian matrix of system (3) is given by

$$J_{3}=\left[\begin{array}{cc} 0.828104\; & -0.386707\\ 0.444512\; & 1. \end{array}\right].$$

Additionally, the characteristic equation from $J_{3}$ is given as

(35) $$\varrho^{2}-1.8281041990948645\varrho +1=0.$$

**Figure 5:**
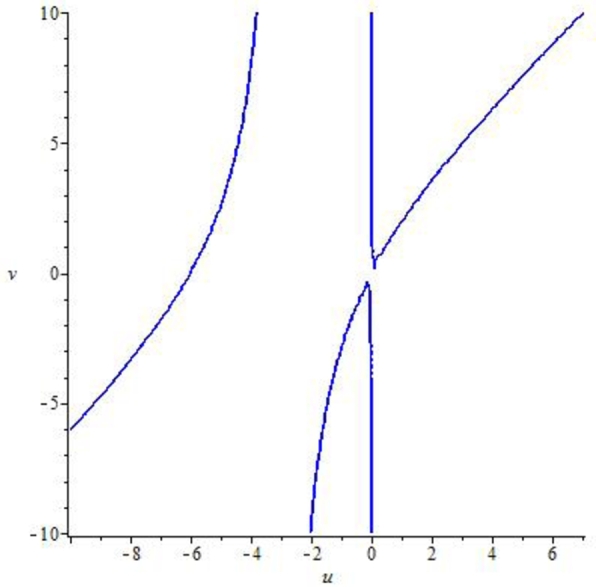
Implicit plot for $\overset{˜}{X}(u,v)$ for *a* = 0.8559,*r* = 3.0609,*β* = 4.8959,*k* = 1.258,*α* = 3.499,*ρ* = 2.9,*δ* = 0.47122,*m* = 0.9, *p*_0_ = 0.26452580082377936,*z*_0_ = 0.7740415103508146 and *η* ∈ (0,1].

**Figure 6:**
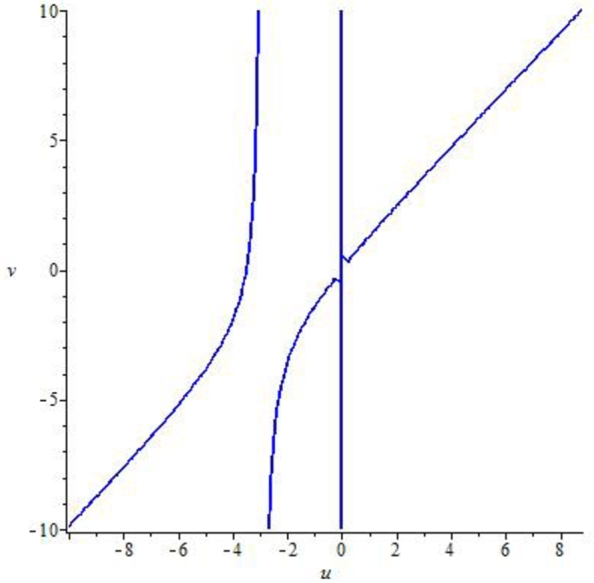
Implicit plot for $\overset{˜}{Y}(u,v)$ for *a* = 0.8559,*r* = 3.0609,*β* = 4.8959,*k* = 1.258,*α* = 3.499,*ρ* = 2.9,*δ* = 0.47122,*m* = 0.9, *p*_0_ = 0.26452580082377936,*z*_0_ = 0.7740415103508146 and *η* ∈ (0,1].

The complex roots for (35) are $\xi_{1}=0.91405209954+0.40559679i$, and $\varrho_{2}=0.9140520995474326-0.4055967942i$ with $|\varrho_{1}|=|\varrho_{2}|=1$. Moreover, by performing some numerical manipulation (Figure 5, Figure 6), we get

$$h_{1}(x,y)=0.2350455474+0.7429701869\;x-0.3867065311\;y-0.6597438115\;x^{2}+0.3451424723\;xy+0.2128121676\;x^{3}-0.3080458090\;x^{2}y,$$

and

$$h_{2}(x,y)=0.7740415105+0.4930744310\;x-0.0000000002808690455\;y-0.4400777184\;x^{2}+0.6370129046\;xy+0.3927772089\;x^{3}-0.5685453722\;x^{2}y,$$

with

$$\overset{˜}{X}(u,v)=0.03182434464\;u^{3}+0.1191233262\;u^{2}\left(0.1710819135\;u-0.4055967942\;v\right)+0.2551272408\;u^{2}+0.3451424723\;\left(0.1710819135\;u-0.4055967942\;v\right)u,$$

and

$$\overset{˜}{Y}(u,v)=0.06942466666\;u^{3}+0.2598670076\;u^{2}\left(0.1710819135\;u-0.4055967942\;v\right)+0.2698683381\;u^{2}+0.7529267725\;\left(0.1710819135\;u-0.4055967942\;v\right)u.$$

**Figure 7:**
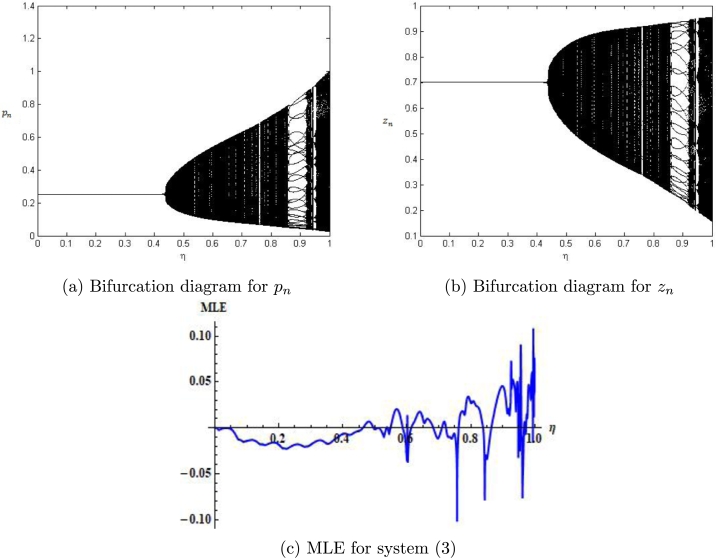
Plots of system (3) for *a* = 0.8559,*r* = 3.0609,*β* = 4.8959,*k* = 1.258,*α* = 3.499,*ρ* = 2.9,*δ* = 0.47122,*m* = 0.9, *p*_0_ = 0.26452580082377936,*z*_0_ = 0.7740415103508146 and *η* ∈ (0,1].

**Figure 8:**
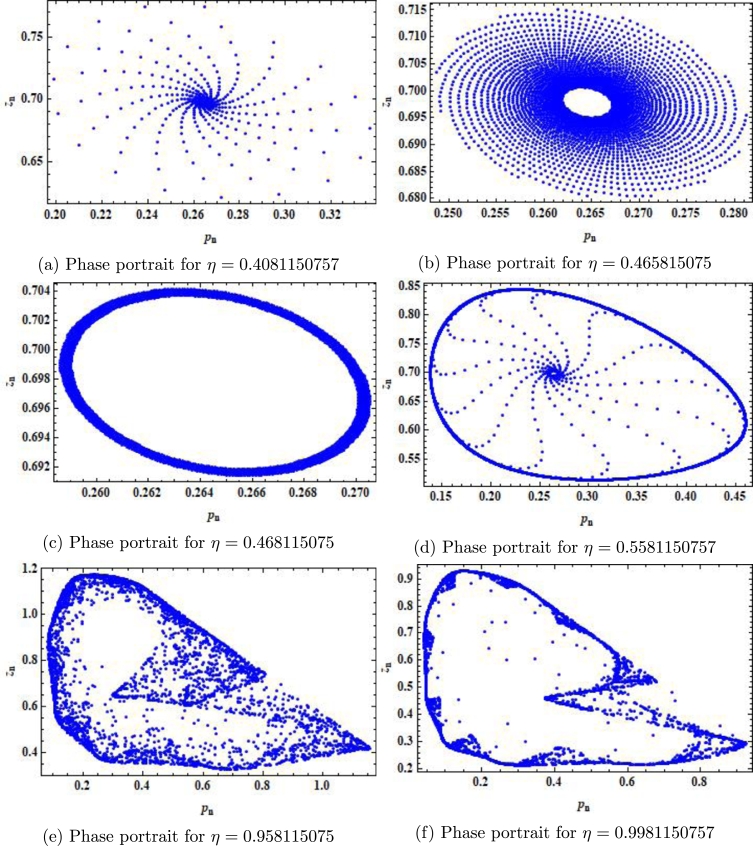
Phase portraits of system (3) for *a* = 0.8559,*r* = 3.0609,*β* = 4.8959,*k* = 1.258,*α* = 3.499,*ρ* = 2.9,*δ* = 0.47122,*m* = 0.9, *p*_0_ = 0.26452580082377936,*z*_0_ = 0.7740415103508146 and *η* ∈ (0,1].

In this case the bifurcation diagrams and MLE are shown in Fig. 7 (a)-(c). Taking a=0.8559,r=3.0609,β=4.8959,k=1.258,α=3.499,ρ=2.9,δ=0.47122,m=0.9, $p_{0}=0.26452580082377936,z_{0}=0.7740415103508146$, phase portraits for system (3) are shown in Fig. 8 (a)-(f) for η=0.4081150757, 0.438115075,0.468115075, 0.5581150757,0.958115075 and η=0.9981150757 respectively. Finally, we have

$$\xi_{20}=\frac{1}{8}\left[{\overset{˜}{X}}_{xx}-{\overset{˜}{X}}_{yy}+2{\overset{˜}{Y}}_{xy}+i\left({\overset{˜}{Y}}_{xx}-{\overset{˜}{Y}}_{yy}-2{\overset{˜}{X}}_{xy}\right)\right]=0.00219755+0.134667i,$$

$$\xi_{11}=\frac{1}{4}\left[{\overset{˜}{X}}_{xx}+{\overset{˜}{X}}_{yy}+i\left({\overset{˜}{Y}}_{xx}+{\overset{˜}{Y}}_{yy}\right)\right]=0.157087+0.19934i,$$

$$\xi_{02}=\frac{1}{8}\left[{\overset{˜}{X}}_{xx}-{\overset{˜}{X}}_{yy}-2{\overset{˜}{Y}}_{xy}+i\left({\overset{˜}{Y}}_{xx}-{\overset{˜}{Y}}_{yy}+2{\overset{˜}{X}}_{xy}\right)\right]=0.15489+0.064673i,$$

$$\xi_{21}=\frac{1}{16}\left({\overset{˜}{X}}_{xxx}+{\overset{˜}{X}}_{xyy}+{\overset{˜}{Y}}_{xxy}+{\overset{˜}{Y}}_{yyy}\right)+\frac{i}{16}\left({\overset{˜}{Y}}_{xxx}+{\overset{˜}{Y}}_{xyy}-{\overset{˜}{X}}_{xxy}-{\overset{˜}{X}}_{yyy}\right)=0.00640142+0.0487457i,$$

and

$$Ϝ={\left(\left[-Re\left(\frac{(1-2\varrho_{1})\varrho_{2}^{2}}{1-\varrho_{1}}\xi_{20}\xi_{11}\right)-\frac{1}{2}|\xi_{11}{|}^{2}-|\xi_{02}{|}^{2}+Re(\varrho_{2}\xi_{21})\right]\right)}_{\overset{¯}{\eta }=0}=-0.11264980623299635\neq 0.$$

Therefore, the condition for the existence of Neimark-Sacker bifurcation is satisfied (see Theorem 4.1).

Example 7.3 We choose a=0.9559,r=2.9609,β=3.08959,k=1.258,α=3.499,ρ=2.7,δ=0.57122,m=0.33, $p_{0}=1.26452580082377936,z_{0}=0.07740415103508146$ and η∈(0,1]. Then, the mathematical system (3) experiences the period-doubling bifurcation whenever the bifurcation parameter *η* certainly passes through η=0.73681150757612096. Furthermore, for these values the jacobian matrix of system (3) is given by

$$J_{3}=\left[\begin{array}{cc} 197.876\; & -45.7371\\ -4.30368\; & 1. \end{array}\right].$$

Additionally, the characteristic equation from $J_{3}$ is given as

(36) $$\varrho^{2}-198.876\varrho +1.03853=0.$$

**Figure 9:**
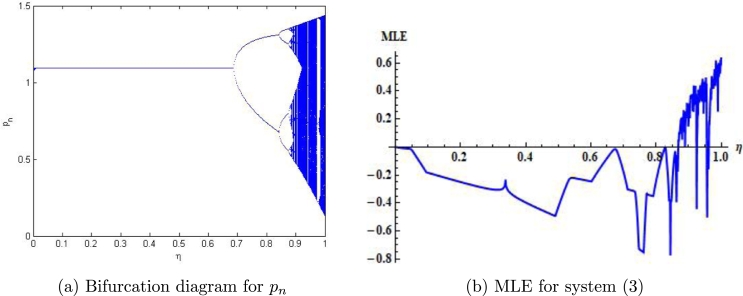
Plots of system (3) for *a* = 0.9559,*r* = 2.9609,*β* = 3.08959,*k* = 1.258,*α* = 3.499,*ρ* = 2.7,*δ* = 0.57122,*m* = 0.33, *p*_0_ = 1.26452580082377936,*z*_0_ = 0.07740415103508146 and *η* ∈ (0,1].

The roots for (36) are $\varrho_{1}=-1$, and $\varrho_{2}=199.87637628645194$. Moreover, the bifurcation diagram for $p_{n}$ and MLE are shown in Fig. 9 (a)-(b).

**Figure 10:**
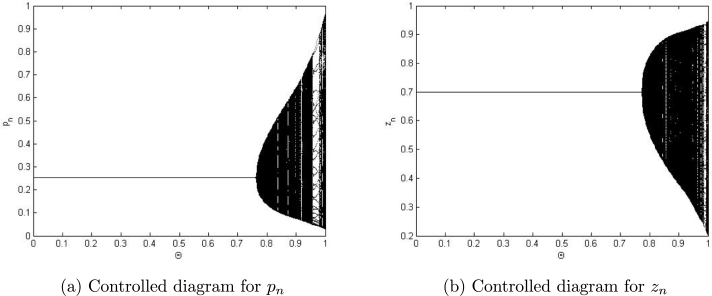
Plots of system (34) for *a* = 0.8559,*r* = 3.0609,*β* = 4.8959,*k* = 1.258,*α* = 3.7799,*ρ* = 2.9,*δ* = 0.47122,*m* = 0.9, *p*_0_ = 0.26452580082377936,*z*_0_ = 0.7740415103508146 and *η* = 0.4681150757612096 for Θ ∈ (0,1].

**Figure 11:**
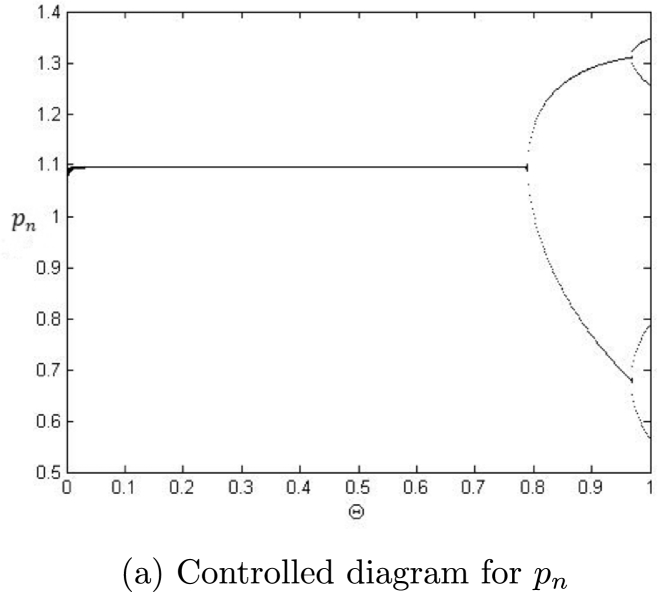
Plots of system (34) for *a* = 0.9559,*r* = 2.9609,*β* = 3.08959,*k* = 1.258,*α* = 3.499,*ρ* = 2.7,*δ* = 0.57122,*m* = 0.33, *p*_0_ = 1.26452580082377936,*z*_0_ = 0.07740415103508146 and *η* = 0.4681150757612096 for Θ ∈ (0,1].

Example 7.4 We choose a=0.8559,r=3.0609,β=4.8959,k=1.258,α=3.7799,ρ=2.9,δ=0.47122,m=0.9, $p_{0}=0.26452580082377936,z_{0}=0.7740415103508146$ and η=0.4681150757612096. Then, the controlled system (34) experiences the Neimark-Sacker bifurcation whenever the controlled parameter Θ certainly passes through the value Θ=0.7740415103508146. Moreover, bifurcation diagrams for system (34) are shown in Fig. 10 (a)-(b). In addition, by taking a=0.9559,r=2.9609,β=3.08959,k=1.258,α=3.499,ρ=2.7,δ=0.57122,m=0.33, $p_{0}=1.26452580082377936,z_{0}=0.07740415103508146$, the controlled system (34) experiences the period-doubling bifurcation whenever the controlled parameter Θ certainly passes through Θ=0.8040415103508146. Moreover, bifurcation diagram for system (34) is shown in Fig. 11. Hence, it can be seen that by applying the generalized hybrid control technique, stability of system (3) can be regained for maximum range of control parameter.

## 8 Conclusions

In our work, we have briefly explained the dynamics of a continuous-time phytoplankton-zooplankton model [28]. Initially, the model taken from [28] is modified by considering the situation that some outer toxic substances are infecting the phytoplankton population. Furthermore, these poisonous substances have an accelerating progression compared to the phytoplankton inhabitants. Then, we have obtained a discrete-time type of model (2) by using Euler's forward method. According to Strogatz [32], chaos occurs in a continuous system when it is at least 3-dimensional. Therefore, it is clear that chaos ceased to exist in the system (2). However, in the case of a discrete-time map, chaos can be observed in 1-dimension. Hence, there is a chance for the existence of chaos in the 2-dimensional discrete-time phytoplankton-zooplankton model. In addition, if chaos does not exist after the discretization then the discretized system is said to be dynamically consistent. Moreover, our main aim is to study a consistent counterpart of the system (2) such that there is a minimal change in the dynamical behaviour of the discretized system compared to the original continuous system. Hence, using the nonstandard finite difference scheme, we have obtained a consistent counterpart (4) of the system (2). The boundedness of every positive solution (4) is discussed. The local asymptotic stability of obtained mathematical system (4) is discussed about each of its fixed points. Moreover, to show the complex behaviour in the mathematical system (4), the presence of Neimark-Sacker bifurcation about a positive fixed point is discussed (see Fig. 3 (a)-(c)). It is shown that the system (4) is dynamically consistent, and there is no chance for the existence of period-doubling bifurcation for system (4) (see Theorem 3.1). The existence of period-doubling bifurcation for a unique positive fixed point of the system (3) is shown mathematically, and some exceptional numerical examples are provided (see Fig. 9 (a)-(b)). It is demonstrated numerically that the system (3) experiences the Neimark-Sacker bifurcation (see Fig. 7 (a)-(c)) and period-doubling bifurcation for an extensive range of stepsize *η*. From Fig. 8 (e) and Fig. 8 (f), the existence of chaos in the system (3) can be seen, which proves the inconsistency of Euler's method (see Fig. 8 (e)-(f)). Finally, Neimark-Sacker bifurcation and period-doubling bifurcation are effectively controlled by using a generalized hybrid method. It is shown that when the generalized hybrid method is applied to the system (3), it regains the system's stability (3) for the maximum range of control parameters (see Fig. 10 (a)-(b)). In addition, the generalized technique of control is comparatively more effective than the hybrid method [49]. It is centred on response control and brings back the system's stability for an extensive range of parameters (see Fig. 11). Moreover, from a numerical study, it is realized that the modified hybrid control methodology is appropriate for controlling the Neimark-Sacker bifurcation and chaos.

## Declarations

### Author contribution statement

Muhammad Salman Khan: Analyzed and interpreted the data; Wrote the paper.

Maria Samreen: Contributed reagents, materials, analysis tools or data.

J.F. Gomez-Aguilar: Analyzed and interpreted the data; Wrote the paper.

Eduardo Perez-Careta: Analyzed and interpreted the data; Contributed reagents, materials, analysis tools or data; Wrote the paper.

### Funding statement

This research did not receive any specific grant from funding agencies in the public, commercial, or not-for-profit sectors.

### Data availability statement

No data was used for the research described in the article.

### Declaration of interests statement

The authors declare no competing interests.

### Additional information

No additional information is available for this paper.
